# Publisher Correction: Tig1 regulates proximo-distal identity during salamander limb regeneration

**DOI:** 10.1038/s41467-022-32302-3

**Published:** 2022-08-03

**Authors:** Catarina R. Oliveira, Dunja Knapp, Ahmed Elewa, Tobias Gerber, Sandra G. Gonzalez Malagon, Phillip B. Gates, Hannah E. Walters, Andreas Petzold, Hernan Arce, Rodrigo C. Cordoba, Elaiyaraja Subramanian, Osvaldo Chara, Elly M. Tanaka, András Simon, Maximina H. Yun

**Affiliations:** 1 Technische Universität Dresden, CRTD/Center for Regenerative Therapies Dresden, Dresden, Germany; 2 Department of Cell and Molecular Biology, Karolinska Institute, Stockholm, Sweden; 3 European Molecular Biology Laboratory (EMBL), Heidelberg, Germany; 4 Institute of Structural and Molecular Biology, University College London, London, UK; 5 Biomedical Research Institute, Foundation for Research and Technology, University of Ioannina Campus, 45115 Ioannina, Greece; 6 Systems Biology Group, Institute of Physics of Liquids and Biological Systems, National Scientific and Technical Research Council (CONICET) and University of La Plata, La Plata, Argentina; 7 Instituto de Tecnología, Universidad Argentina de la Empresa (UADE), Buenos Aires, Argentina; 8 Technische Universität Dresden, Center for Information Services and High Performance Computing ZIH, Dresden, Germany; 9 Institute of Molecular Pathology, Vienna Biocenter, Vienna, Austria; 10 Max Planck Institute for Molecular Cell Biology and Genetics, Dresden, Germany

**Keywords:** Regeneration, Body patterning, Pattern formation

Correction to: *Nature Communications* 10.1038/s41467-022-28755-1, published online 03 March 2022

The original version of this Article contained errors in Figs. 2 and 5. In Fig. 2, the graphs were inadvertently displayed as line graphs (2a) and bar charts (2b) rather than dot plots. Figure 2b and 2c were mislabeled as 2c and 2b; this has now been corrected. In Fig. 5c the last row incorrectly read “X/X X.0” rather than “6/9 66.7”. This has now been corrected in the PDF and HTML versions of the Article.

