# Supplementary material for: Tig1 regulates proximo-distal identity during salamander limb regeneration
Source: Nat Commun. 2022 Mar 3;13:1141. doi: 10.1038/s41467-022-28755-1 (PMC8894484; doi:10.1038/s41467-022-28755-1)
Supplement: Supplementary file 1 — Supplementary Information [file 41467_2022_28755_MOESM1_ESM.pdf]

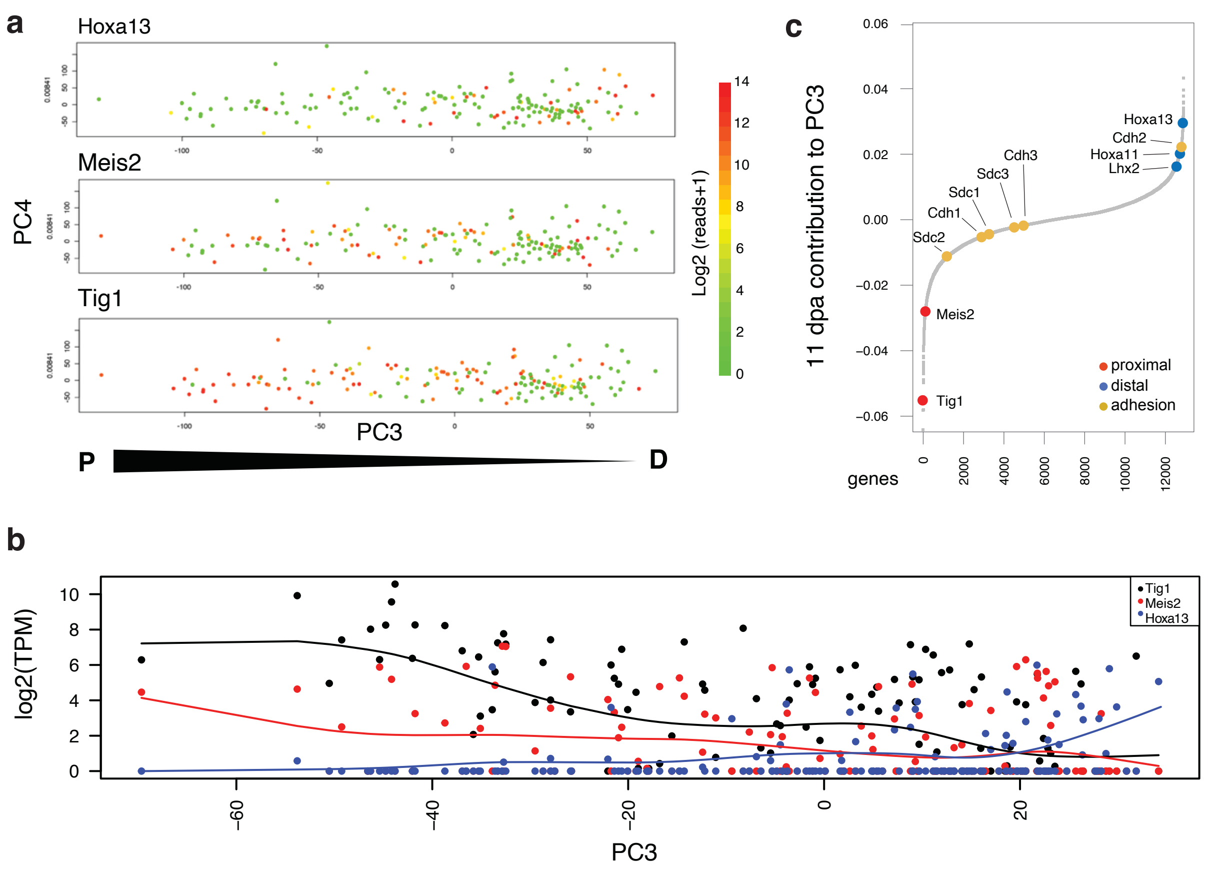

Supplementary Fig. 1. Tig1 expression is segregated along a modelled proximodistal axis of the regenerating limb. (a) Principal component analysis (PCA) (PC3 vs PC4) of individual connective tissue cells at 11 days post amputation (dpa), based on scRNAseq from Gerber et al17. In this dataset PC3 models the proximodistal axis in the regenerating blastema, as exemplified by the expression of Meis2 (proximal) and Hoxa13 (distal). (b) PC3 plotted against log2(transcripts per million) for the indicated genes. (c) Contributions of axolotl genes to PC3. Genes associated with the proximal (red) and distal (blue) axes, or with cell adhesion (yellow) are indicated. This scRNAseq dataset does not contain expression data for Prod1. Note that Tig1 is found at the extreme Meis2+ end of PC3. In this dataset we found 90/163 cells express Tig1, 43/163 express Hoxa13 and 61/163 express Meis2. PC3 explains 1.2% of the variance.

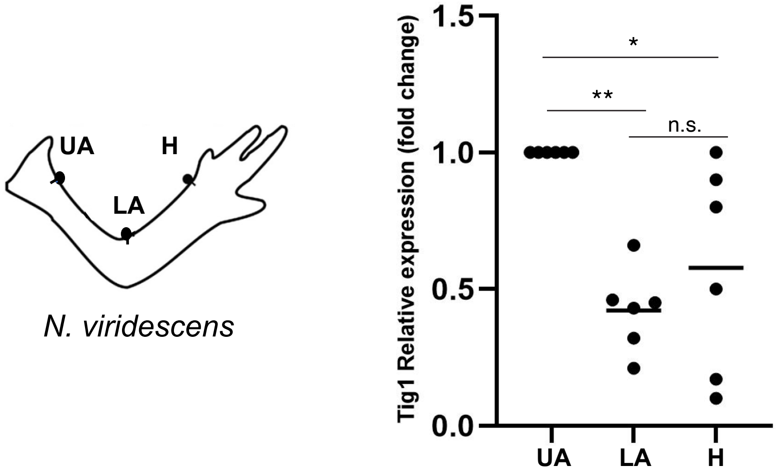

Supplementary Fig. 2. Tig1 is expressed in gradient along the proximo-distal axis in newt limbs. (a) Tig1 expression along the proximo-distal axis of the mature limb of 3-4 years old *Notophthalmus viridescens* newts. Expression is relative to Ef1-α. UA: upper arm; LA: lower arm; H: hand; qRT-PCR (n=6 biologically independent samples). *Adj. p=0.0498, **Adj. p=0.0054 (Kruskal Wallis followed by Dunn's multiple comparison test). Source data are provided as a Source Data file.

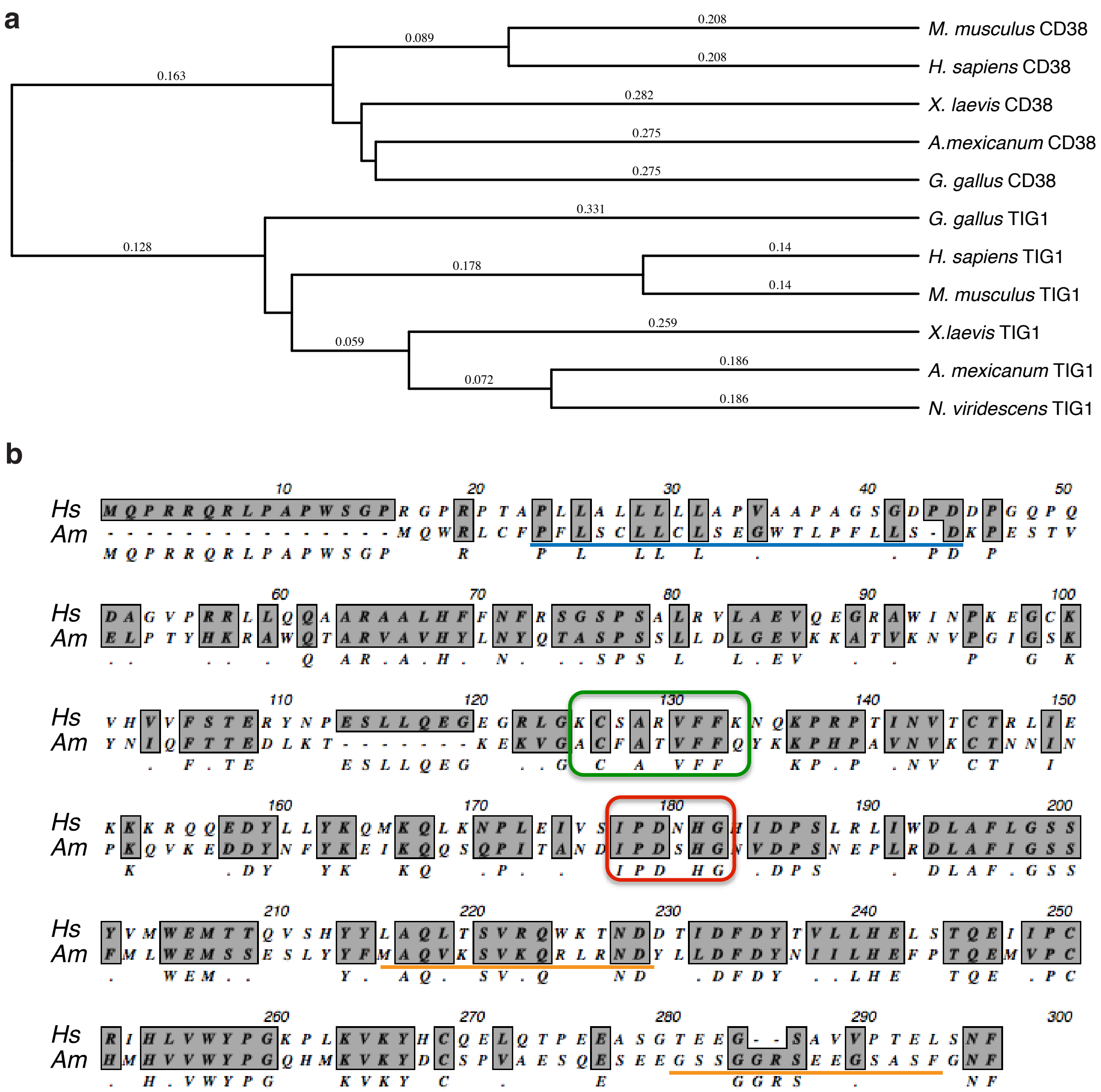

Supplementary Fig. 3. Axolotl TIG1 protein sequence. (a) Phylogenetic tree based on the indicated Tig1 and CD38 protein sequences, built using the unweighted pair group method with arithmetic mean (UPGMA) algorithm. (b) Sequence alignment between axolotl TIG1 and its human counterpart. Axolotl TIG1 exhibits 57% similarity at the aminoacid level, an intact latexin domain (IPDXXG, red square) and a hyaluronic acid binding motif (green square). The peptide sequences selected for subsequent antibody production are underscored in yellow. The hydrophobic tract corresponding to the transmembrane domain is underscored in blue.

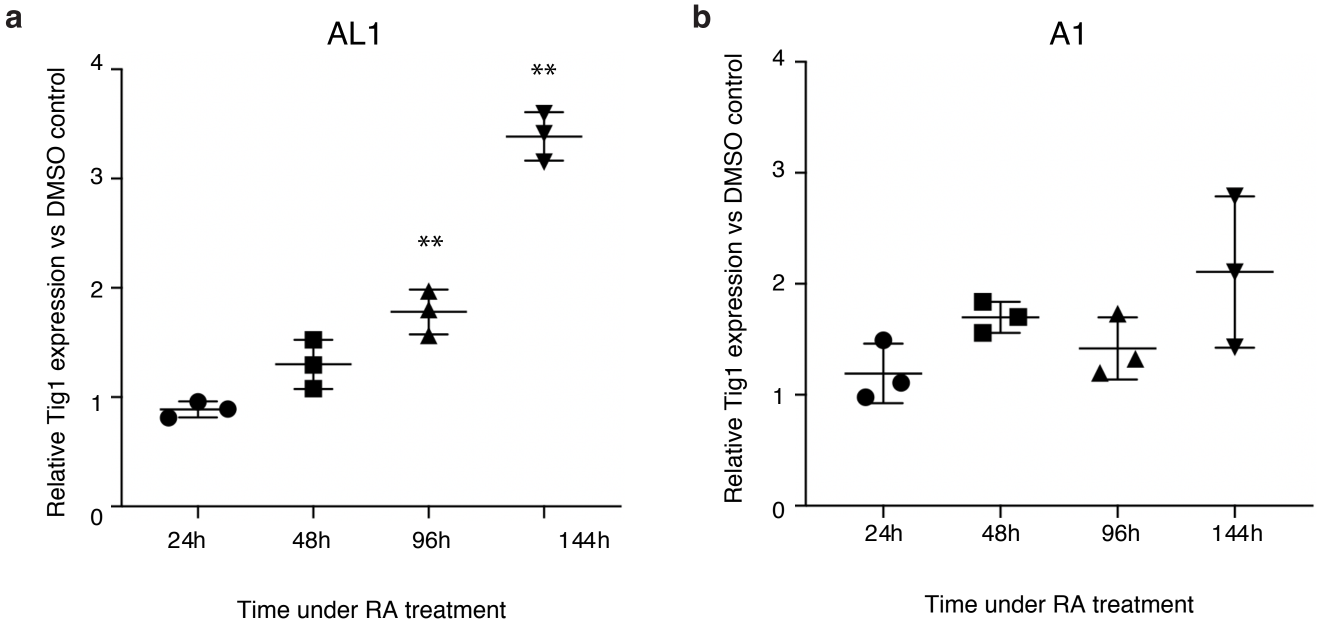

Supplementary Fig. 4. Tig1 is upregulated by RA in cells of connective tissue origin. (a,b) qRT-PCR analysis of Tig1 expression in connective tissue- derived axolotl AL1 cells (a) or muscle-derived newt A1 cells (b). Data represents Tig1 gene expression at the indicated times following treatment with 1x10-7 M RA, relative to treatment with vehicle (DMSO). Results are normalised against Ef1-α (n=3 biologically independent experiments). **p=0.0178 (AL1, 24h vs 96h), p=0.0116 (AL1, 24h vs 144h) (One-way ANOVA followed by Tukey's multiple comparison test). Error bars represent S.E.M. Source data are provided as a Source Data file.

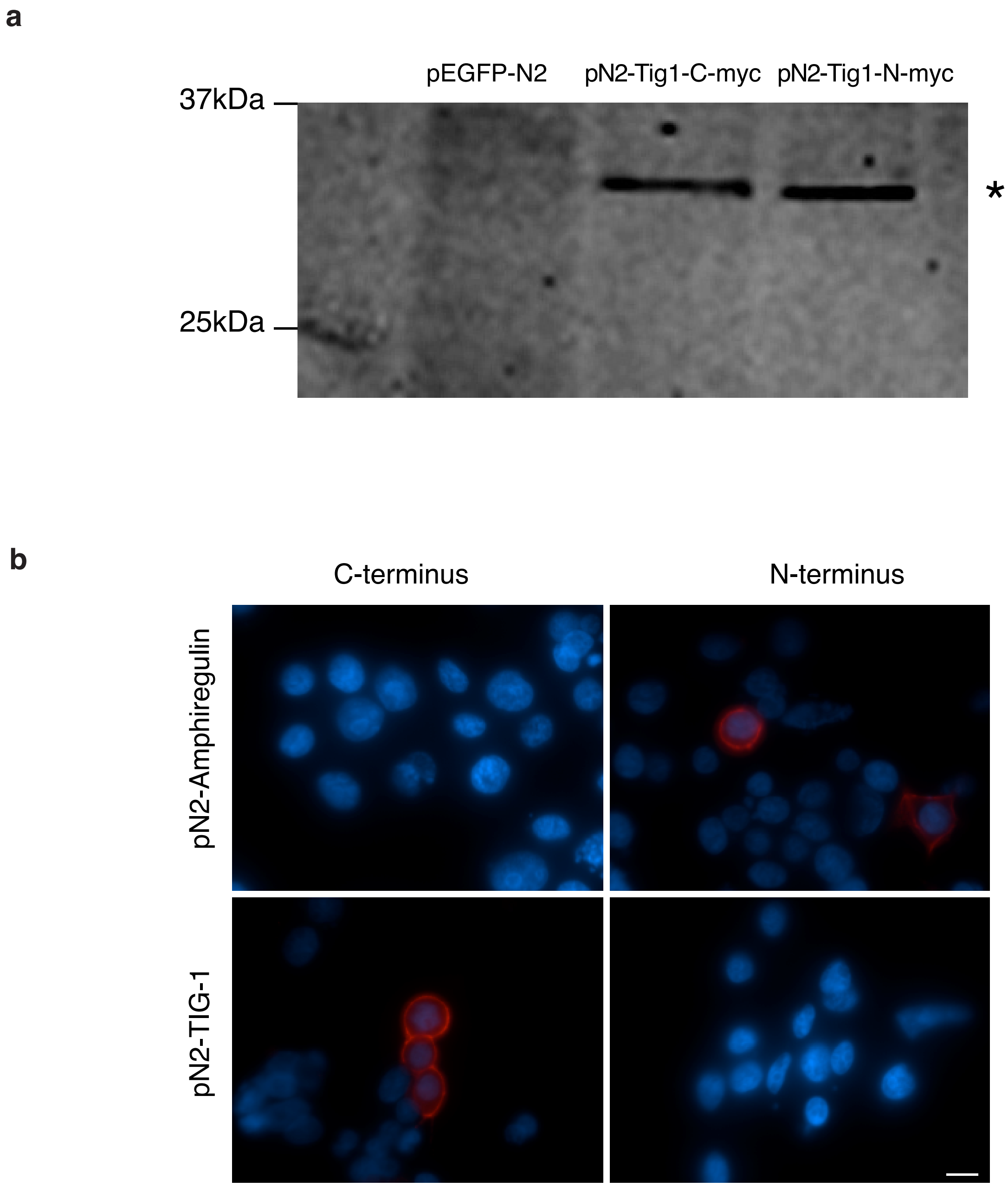

Supplementary Fig. 5. Axolotl TIG1 localises to the cell surface exposing its C-terminus to the extracellular environment. (a) Western blot analysis of myc-tagged protein in *Am*AL1 extracts 48hs after nucleofection with the indicated vectors. Asterisk indicates the band corresponding to the TIG1-myc fusion (31kDa). (b) Representative images of 293T cells 24 hours after transfection with myc-tagged versions of axolotl amphiregulin or TIG1 followed by live cell staining using α-myc antibodies. Scale bar: 20μm. Each experiment was independently repeated 3 times with similar results.

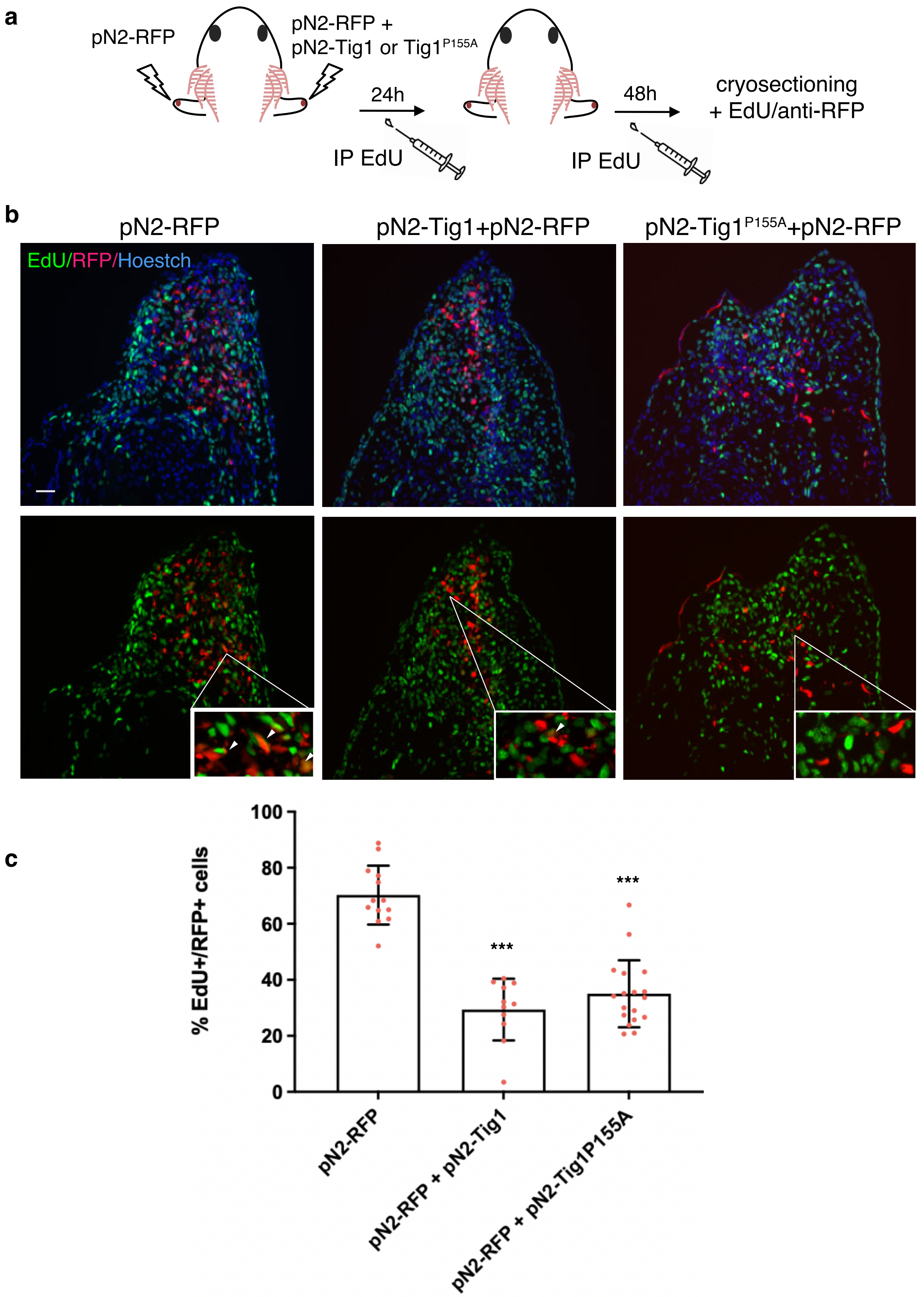

Supplementary Fig. 6. Tig1 overexpression impairs blastema cell proliferation *in vivo*. (a) Schematic of experimental strategy (b) Representative images of axolotl late-bud blastemas following anti-RFP immunohistochemistry and azide-mediated EdU detection, 72h after electroporation with the indicated constructs and after two EdU pulses at 24h and 48h post electroporation (n=5). Insert shows a magnification of the blastema area. Arrowheads indicate EdU+/RFP+ cells. Scale bar: 100µm. (c) Percentage of EdU+ cells among the RFP+ cell population. ***p=4.56E-09 (pN2-RFP vs pN2RFP+pN2-Tig1), p=2.38E-09 (pN2-RFP vs pN2RFP+pN2-Tig1P155A) (two-tailed paired-matched t-test). Error bars indicate S.E.M. n indicates biologically independent samples. Source data are provided as a Source Data file.

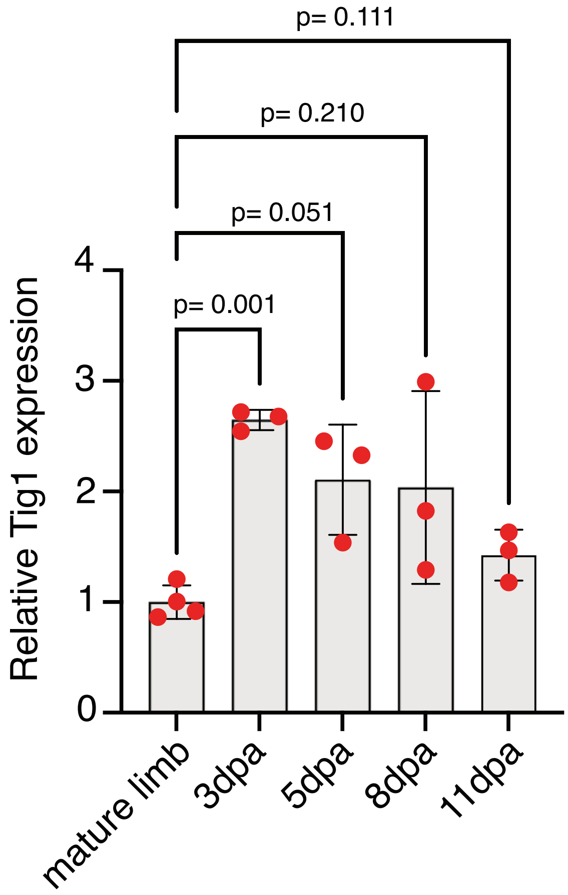

Supplementary Fig. 7. Upregulation of Tig1 expression during early stages of axolotl limb regeneration. Tig1 expression levels in the axolotl upper arm blastema including 500μm of tissue proximal to the amputation plane at the indicated times after amputation as determined by qRT-PCR. Expression is normalised to Rlp4 and visualised as fold change relative to the intact limb (mature). The chart shows the means, standard deviations and adjusted p-values corresponding to Dunnet's multiple comparison test against mature limb following 2-way ANOVA (n=3-4). The individual points represent the means of the technical replicates. Column top indicates mean, error bars indicate S.E.M. n=biologically independent experiments. Source data are provided as a Source Data file.

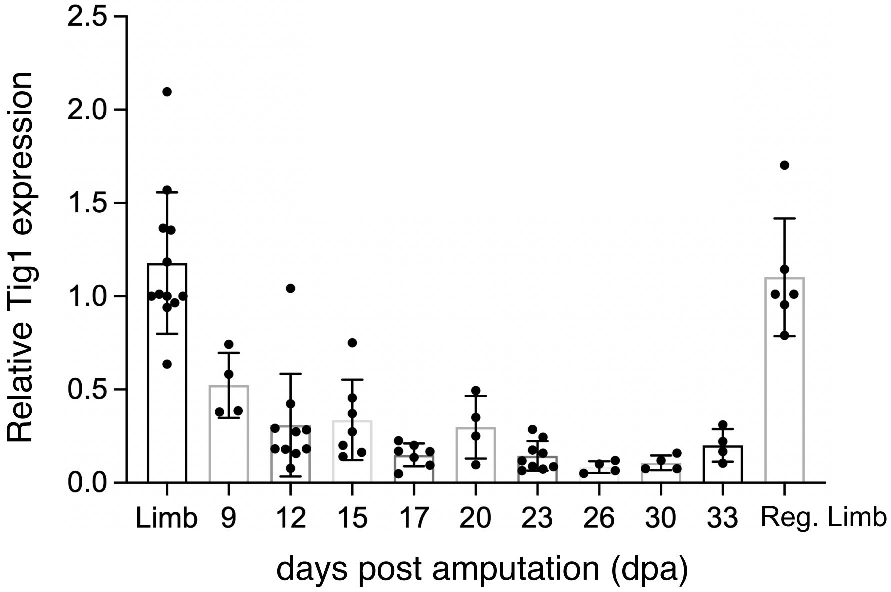

Supplementary Fig. 8. Analysis of Tig1 gene expression during mid and late stages of axolotl limb regeneration. Expression of Tig1 in blastema tissue during axolotl limb regeneration following amputation at the upper arm level, relative to mature limb and normalised against Ef1-α (qRT-PCR, n=4-11). Similar results were obtained normalising against L-27. Adj. p-values: 0.0179 (limb vs 12dpa; 0.0004 (limb vs 17dpa; <0.0001 (limb vs 23dpa); 0.0001 (limb vs 26dpa); 0.0005 (limb vs 30dpa) (Kruskal-wallis test followed by Dunns' multiple comparisons test). Column top indicates mean, error bars indicate S.E.M. n=biologically independent experiments. Source data are provided as a Source Data file.

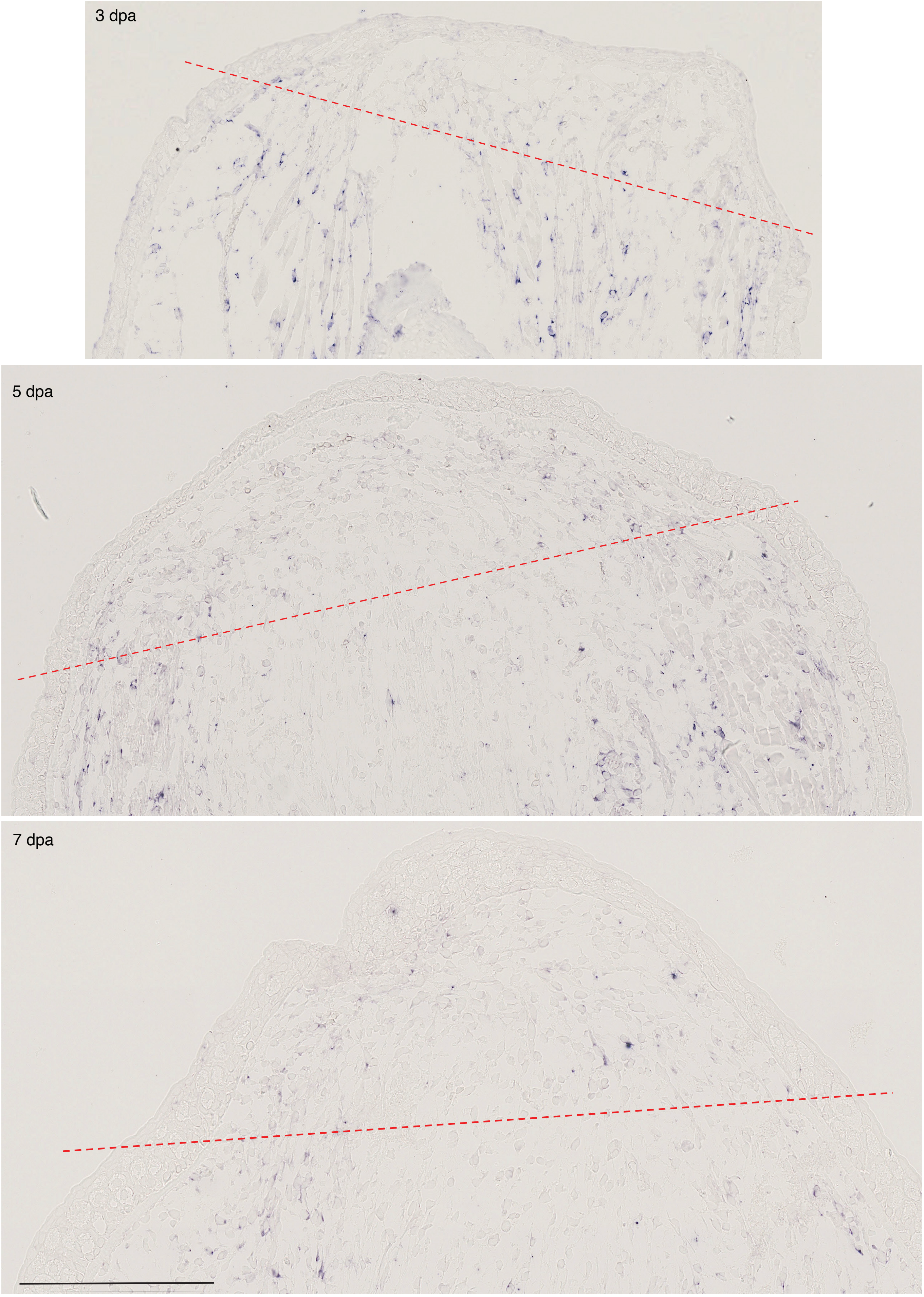

Supplementary Fig. 9. Tig1 gene expression at 3, 5 and 7 days after upper arm amputation. Expression of axolotl Tig1 during lower arm limb regeneration (7.5cm snout-to-tail axolotls) by in situ hybridization. Red dotted line indicates amputation plane. Scale bar: 500μm. Each experiment was independently repeated 4 times with similar results.

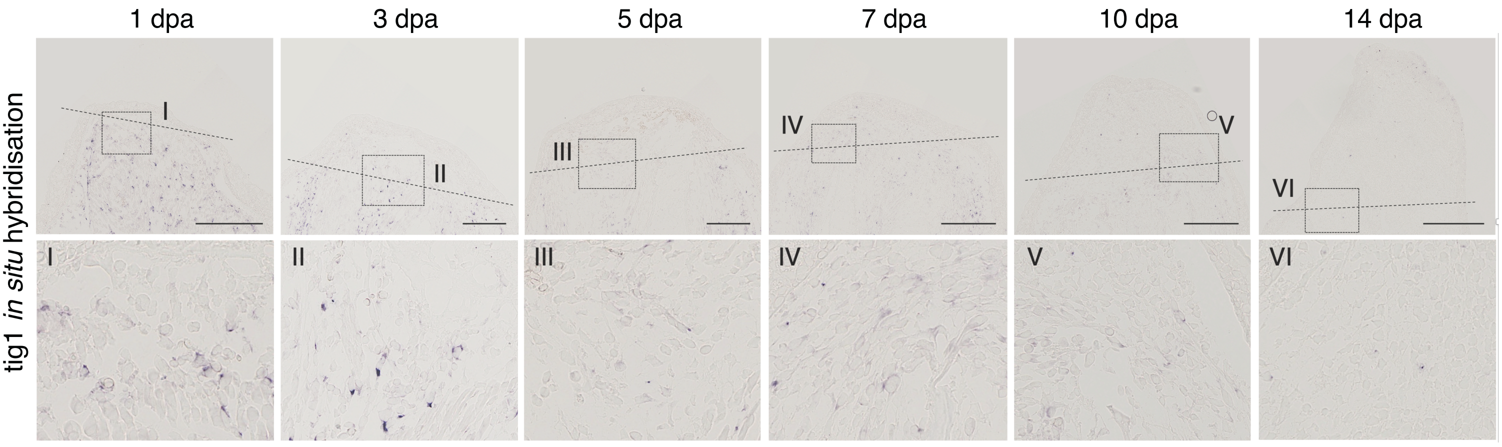

Supplementary Fig. 10. Tig1 gene expression changes during limb regeneration following lower arm amputation. Expression of axolotl Tig1 during lower arm limb regeneration (7.5cm snout-to-tail axolotls) by *in situ hybridization*. Representative images show blastema (upper row) or corresponding magnified area (lower row). Scale bar: 500μm. Scale bar: 500μm.Each experiment was independently repeated 3 times with similar results.

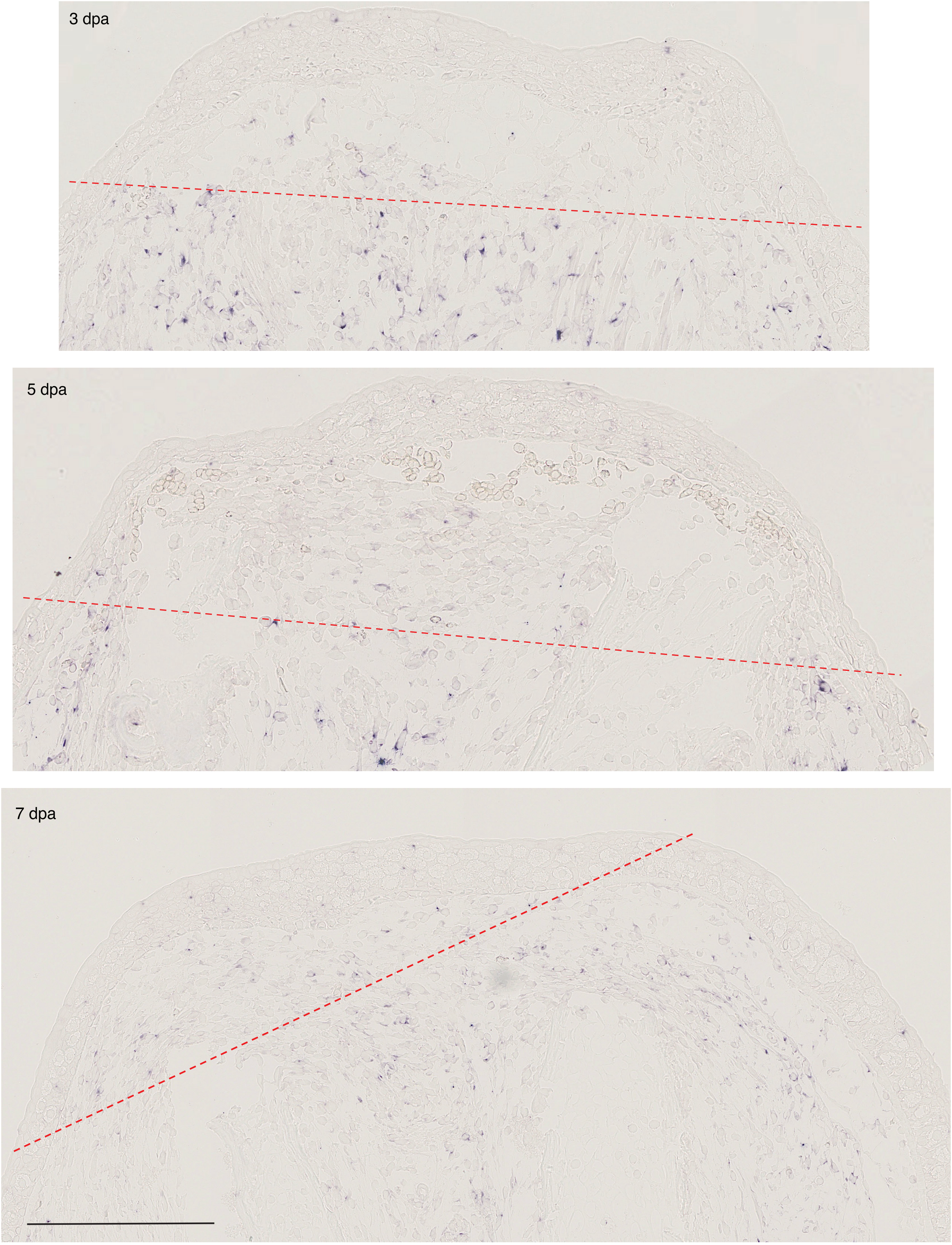

Supplementary Fig. 11. Tig1 gene expression at 3, 5 and 7 days after lower arm amputation. Expression of axolotl Tig1 during lower arm limb regeneration (7.5cm snout-to-tail axolotls) by *in situ hybridization*. Red dotted line indicates amputation plane. Scale bar: 500μm.Each experiment was independently repeated 3 times with similar results.

a

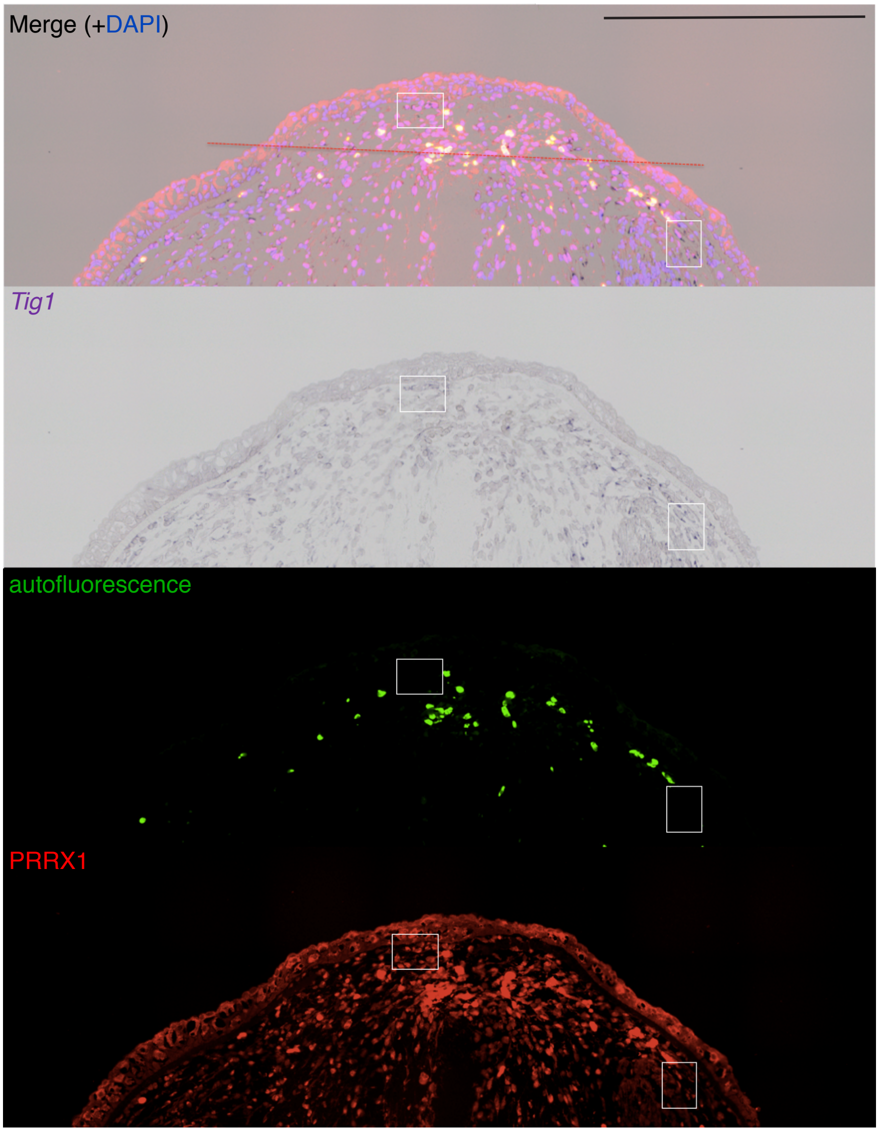

b **c**

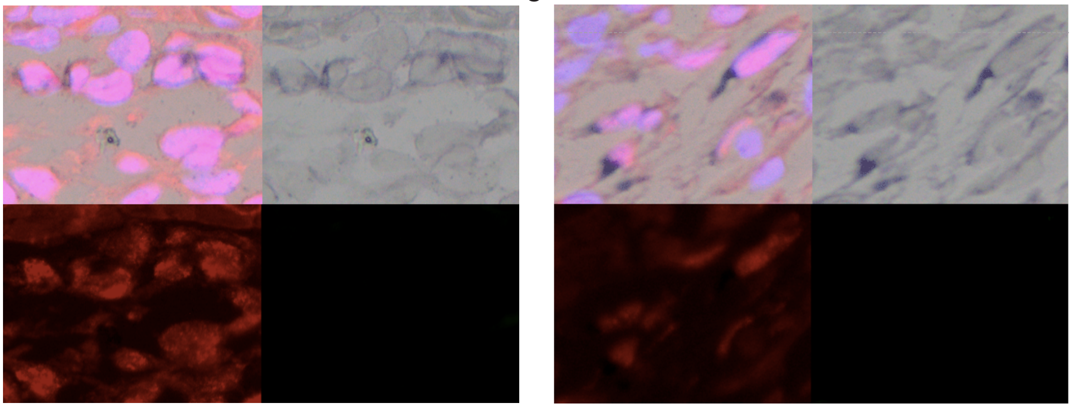

Supplementary Fig. 12. Tig1 gene expression in connective tissue cells during limb regeneration. (a) Representative images of an early blastema (5dpa, upper arm, 7.5cm snout-to-tail axolotls) following Tig1 *in situ hybridization* and anti-PRRX1 staining. Tissue autofluorescence (green) is also shown. Similar results were obtained at 3 and 7 dpa. In all samples analysed, 70-80% of PRRX1+ cells express Tig1. Selected magnified areas are shown in (b) and (c). Scale bar: 500μm. Each experiment was independently repeated 4 times with similar results.

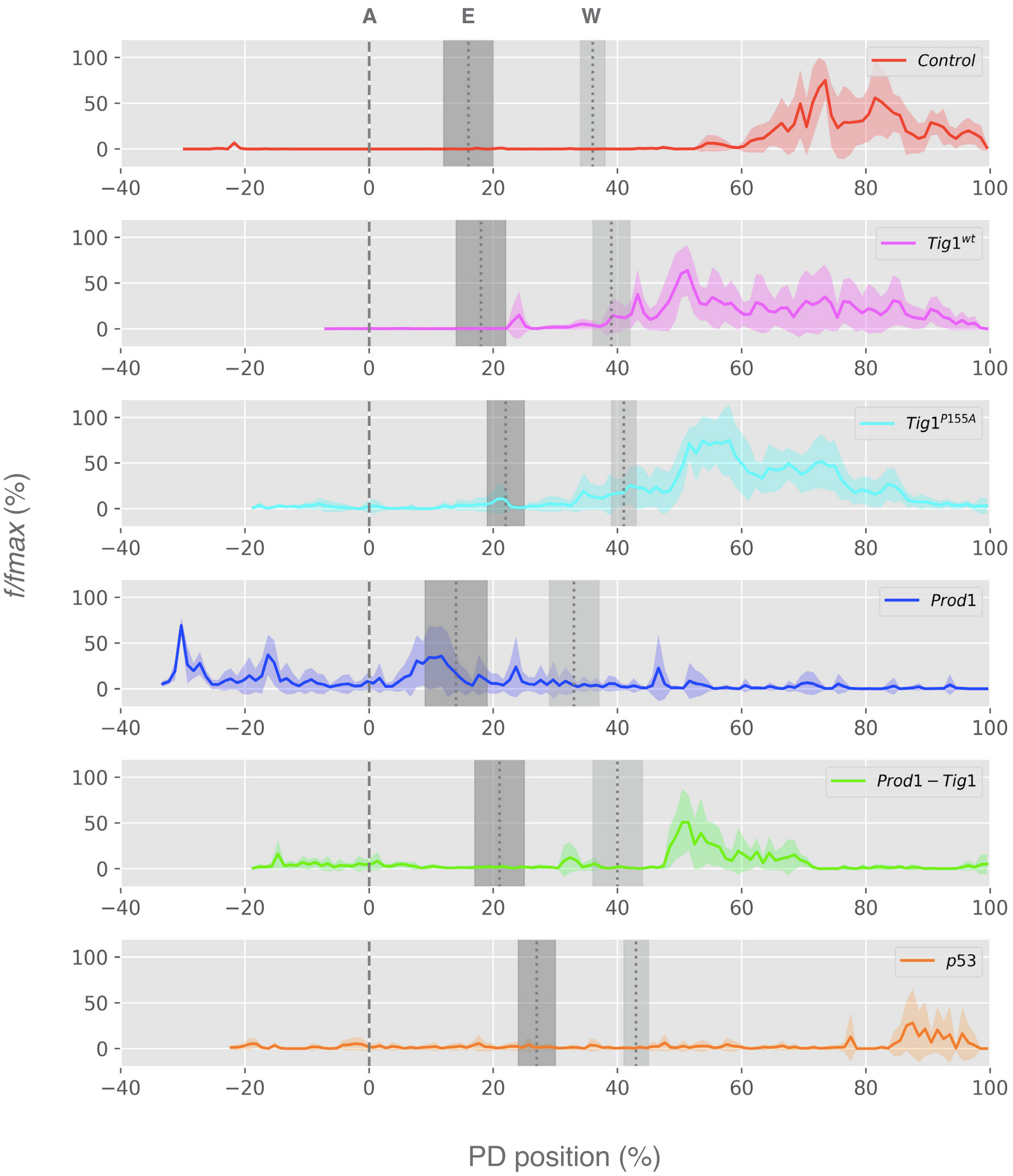

Supplementary Fig. 13. Meandros f/fmax charts per experimental condition. Plots of fluorescence distribution along the PD axis as obtained by Meandros image analysis algorithm are given for each individual condition. Dashed line at PD position=0 indicates the location of the amputation plane (A). Coloured shading indicates standard deviation of f/fmax, coloured center indicates mean. Grey shading indicates standard deviation of the PD position corresponding to elbow (E) or wrist (W). Control (n=3), Tig1wt (n=4), Tig1^{P155A} (n=4), Prod1 (n=5), Prod1-Tig1wt (n=3), p53 (n=3 biologically independent experiments).

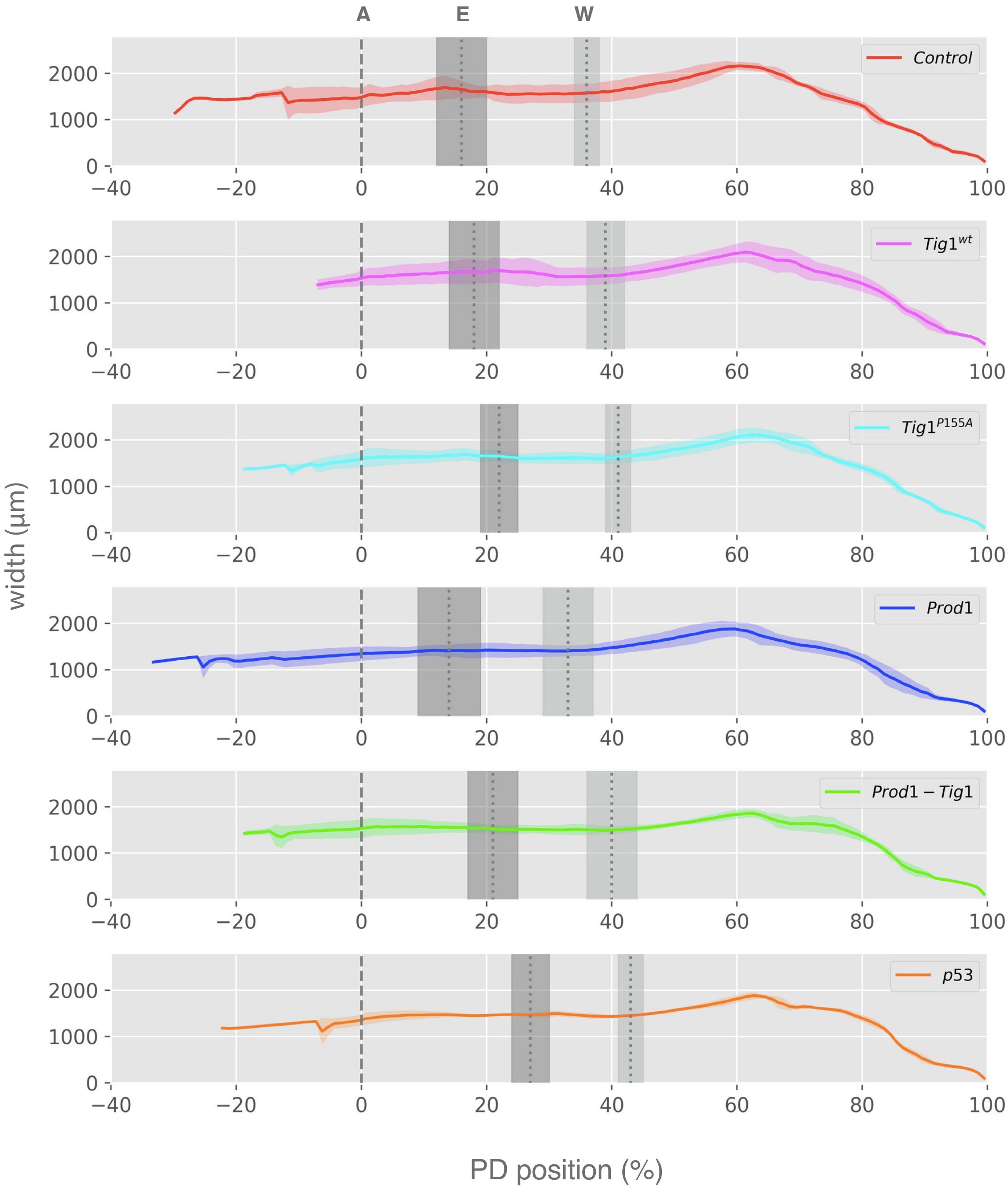

Supplementary Fig. 14. Meandros scaled width analysis along the PD axis. Plots representing the variation of limb width (μm) along the PD axis as obtained by Meandros image analysis algorithm are given for each individual condition. Dashed line at PD position=0 indicates the location of the amputation plane (A). Coloured shading indicates standard deviation of scaled width, coloured center indicates mean. Grey shading indicates standard deviation of the PD position corresponding to elbow (E) or wrist (W).

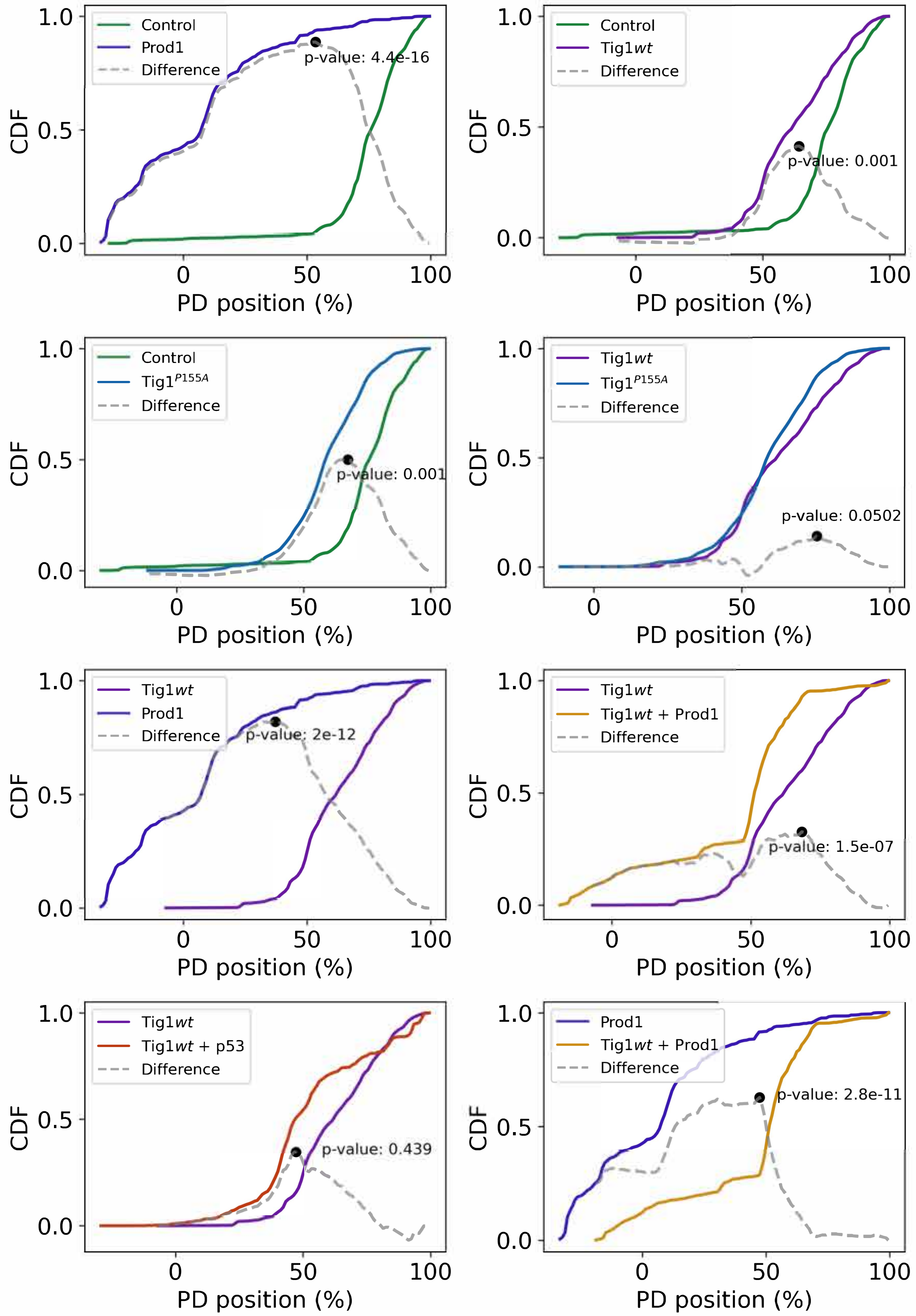

Supplementary Fig. 15. Statistical analysis of Meandros f/fmax profiles. Kolmogorov–Smirnov (two-sample, two-tailed) statistical test comparing the cumulative distribution function (CDF) of the mean f/fmax profiles for the indicated pairs of conditions (in continuous curves). The dashed line represents the distribution of the difference between CDFs and the p-value for each test is indicated.

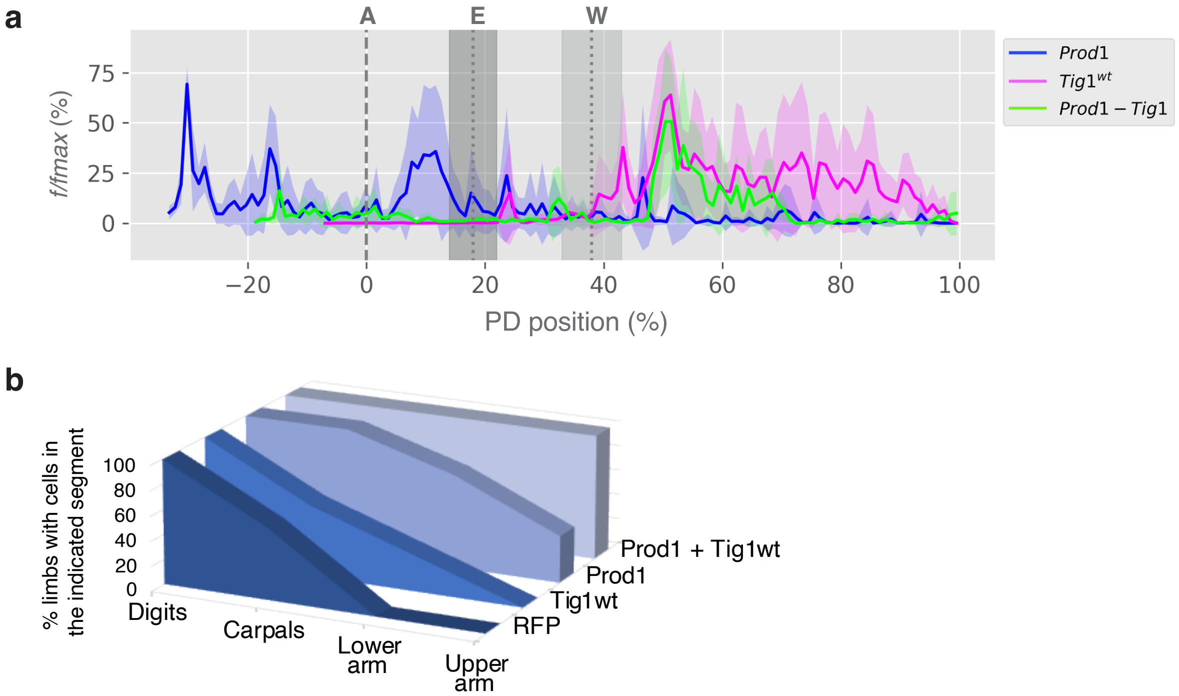

Supplementary Fig. 16. Tig1 and Prod1 have differential effects on distal-to-proximal blastema cell displacement. (a) Distribution of fluorescence intensity along the PD axis for the indicated conditions. Coloured shading indicates standard deviation, coloured center indicates mean. Grey shading indicates standard deviation of the PD position corresponding to elbow (E) or wrist (W). A: amputation plane. (b) Quantification of the number of limbs with electroporated cells in the indicated segments following a displacement assay. Control (n=7), Tig1wt (n=7), Prod1 (n=8) and Prod1-Tig1wt (n=12). Source data are provided as a Source Data file.

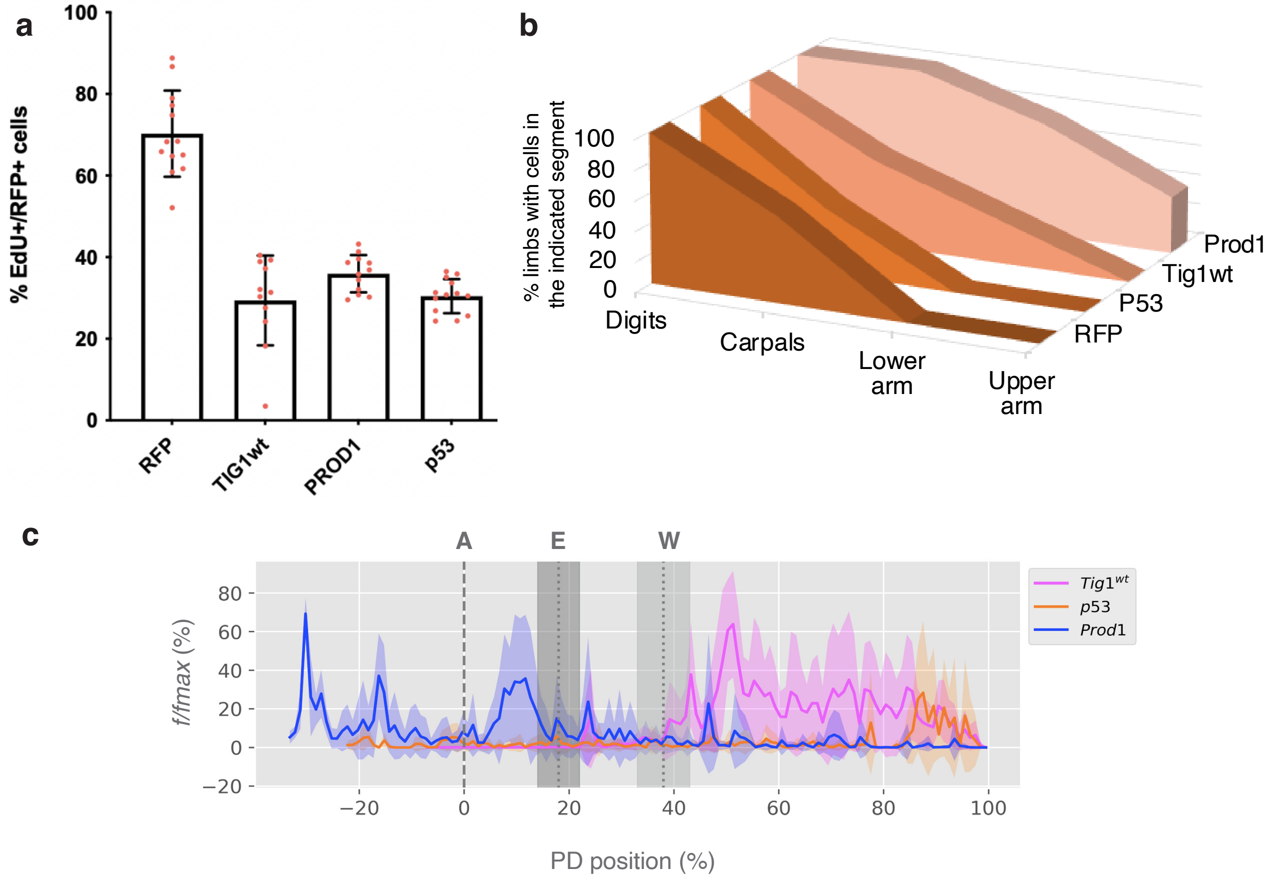

Supplementary Fig. 17. Impairment in cell cycle progression is not sufficient to promote distal-to-proximal blastema cell displacement. (a) Quantification of EdU+/RFP+ blastema cells following anti-RFP immunohistochemistry and azide-mediated EdU detection, 72 hours post electroporation with the indicated pN2 vectors and after two EdU pulses at 24h and 48h post electroporation (n = 5 biologically independent samples). ***p=4.56E-09 (RFP vs TIG1), p=3.55E-10 (RFP vs PROD1), p=4.08E-12 (RFP vs P53) (two-tailed paired-matched t-test). Error bars represent S.E.M. (b) Quantification of the number of animals with electroporated cells in the indicated limb segments following a displacement assay using a control vector (n=7), p53 (n=7), Tig1wt (n=7), and Prod1 (n=8). (c) Distribution of fluorescence intensity along the PD axis for the indicated conditions. Coloured shading indicates standard deviation, coloured center indicates mean. Note that p53 overexpression does not lead to distal-to-proximal displacement. Grey shading indicates standard deviation of the PD position corresponding to elbow (E) or wrist (W). A: amputation plane. Source data are provided as a Source Data file.

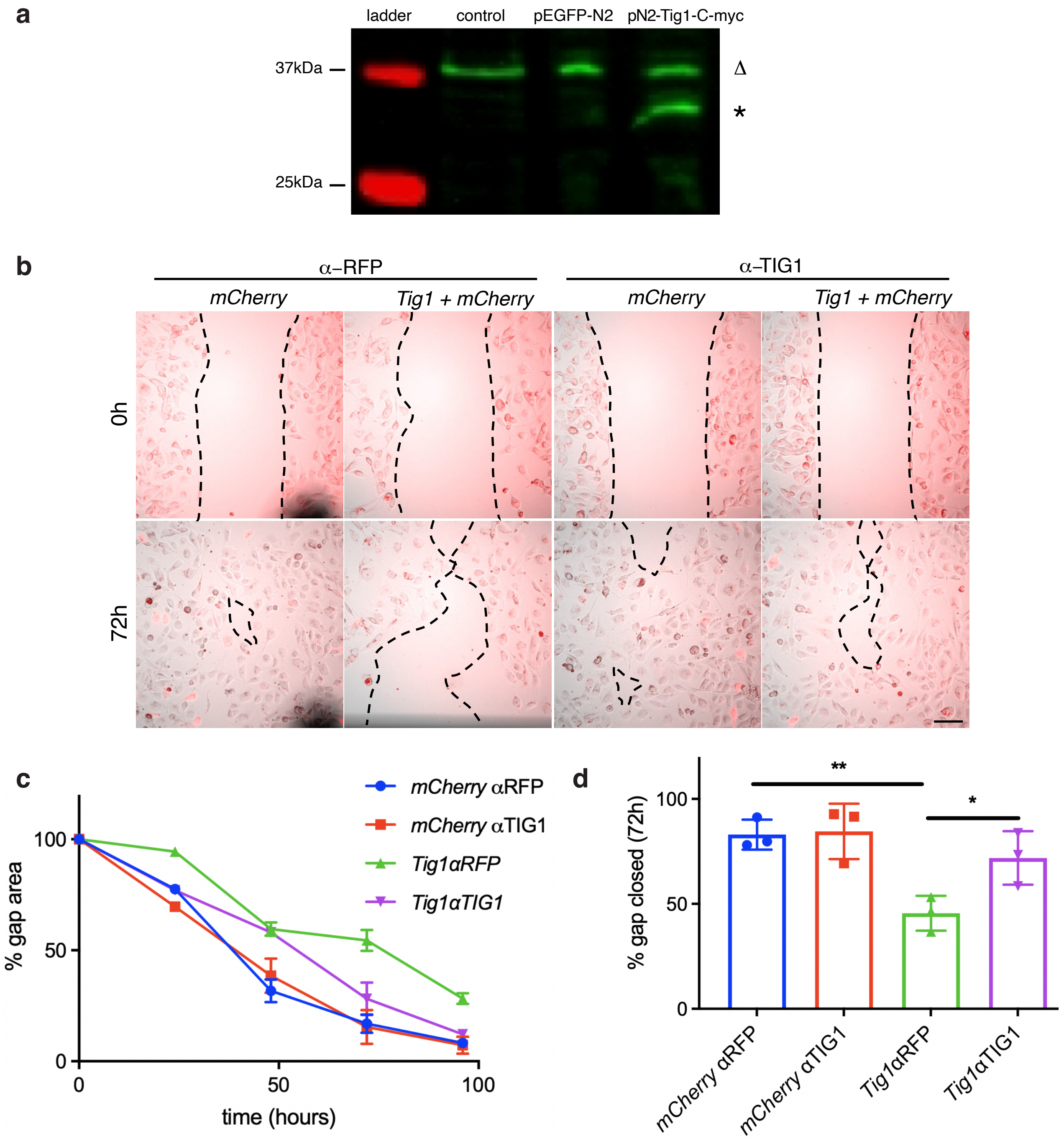

Supplementary Fig. 18. Tig1 levels impact on cell migration. (a) Western blot analysis of 293T cell extracts 48hs after lipofection with the indicated vectors, using the Tig1 antibody mix. Asterisk indicates the band corresponding to the TIG1-myc fusion (31kDa). Triangle indicates an unspecific band, present in all extracts including the untransfected control. (b-d) Scratch assay for evaluation cell migration in AL1 cells. (b) Representative images of AL1 cells. Migration was evaluated for 100hs, starting at 48hs after lipofection with the indicated constructs (0h) and in the presence of either -RFP or -Tig1 antibodies. Scale bar: 200*μ*m. (c) Kinetics of cell migration for the indicated conditions. Cells were imaged every 24 hours and gap closure was quantified as the % cell-free area remaining relative to the 0h timepoint. (d) % gap closure at 72h for the indicated conditions. Note that treatment with -Tig1 antibodies neutralises the negative impact of TIG1 overexpression on cell migration. p-values: *p=0.0398, **p=0.004 (two-tailed, unpaired t-test). Error bars indicate S.E.M. (b-d: n=3 independent biological experiments). Source data are provided as a Source Data file.

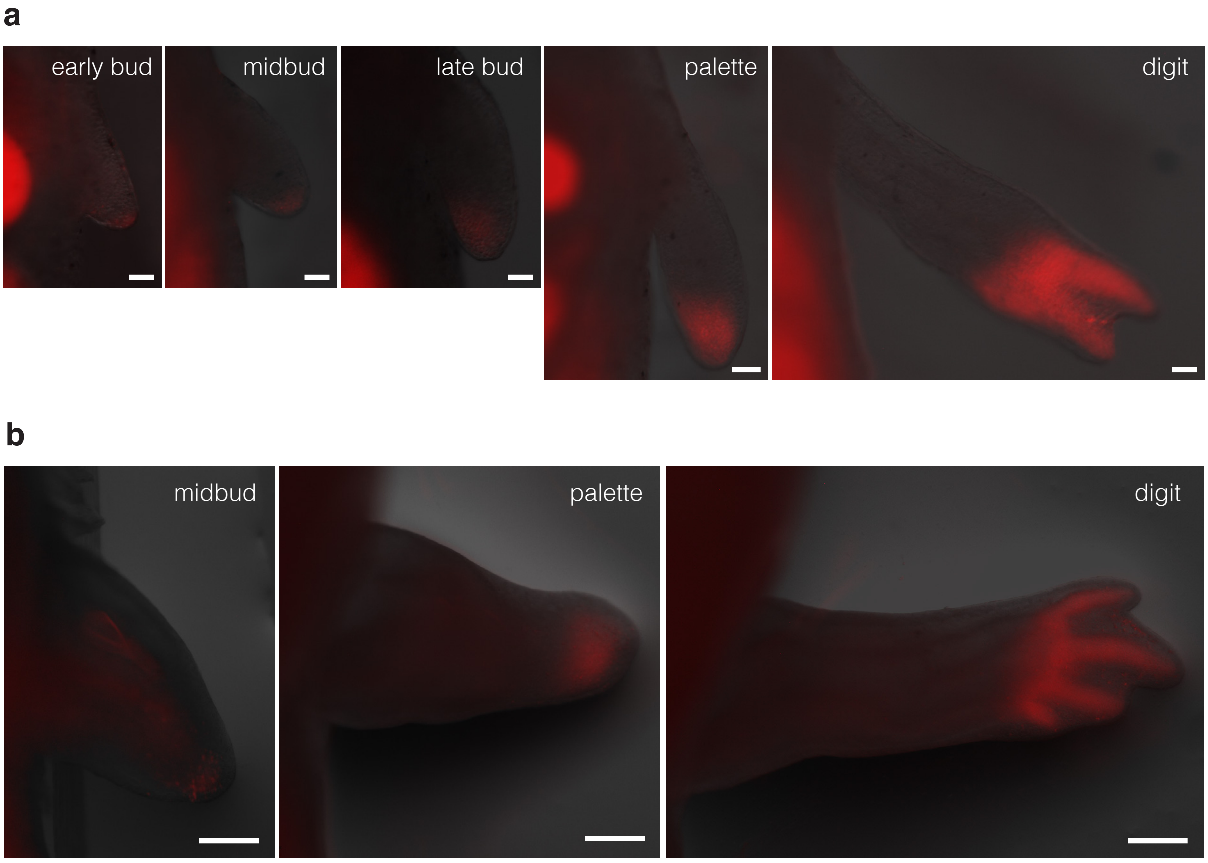

Supplementary Fig. 19. A Hoxa13 knock-in reporter line allows distal cell labelling during salamander limb regeneration. (a) Representative images of limb bud development in the *Hoxa13:Hoxa13-T2a-mCherry* axolotl line. Scale bar: 100μm. (b) Representative images of limb regeneration in *Hoxa13:Hoxa13-T2a-mCherry* axolotls. mCherry fluorescence appears in the distal domain of midbud blastema, and intensifies through the course of regeneration until digit stage. Scale bar: 500μm.

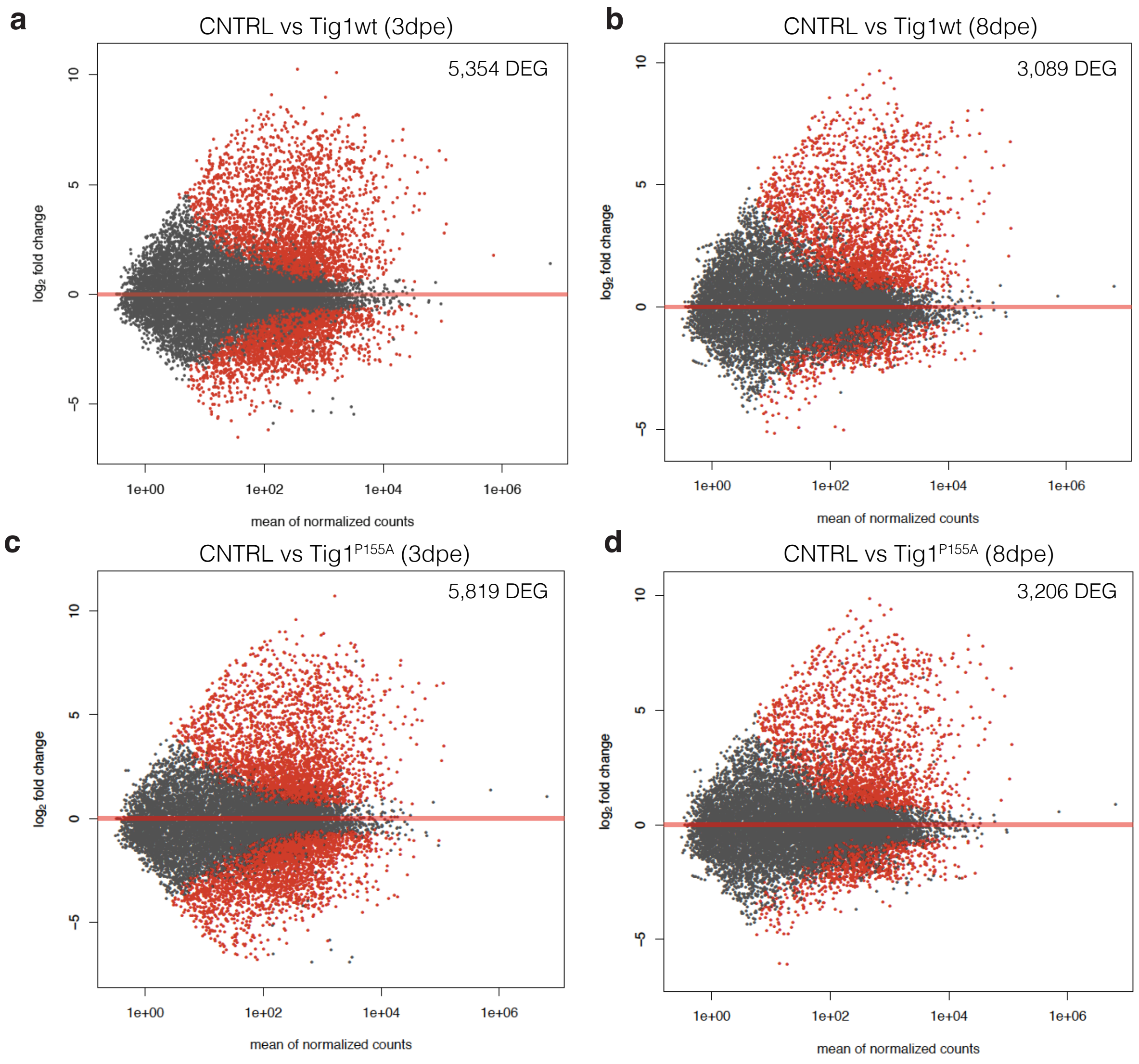

Supplementary Fig. 20. Tig1 overexpression promotes broad transcriptomic changes. (a-d) MA plot representation of differential expression analysis of genes expressed in control distal cells (mCherry+) versus Tig1 (a, b) or Tig1^{P155A} (c, d) overexpressing distal cells (mCherry+/GFP+), at 3 (a,c) or 8 (b, d) days post electroporation. The number of differentially expressed genes (DGE) for each comparison is annotated in the top right corner. Red points mark the genes with significantly increased or decreased expression (FDR<0.05). The number of differentially expressed genes is indicated in the top left corner of each graph.

a

GO Analysis - Cluster B

adj.Pval	nGenes	Pathways
8.50E-90	325	Immune system process
1.40E-80	266	Immune response
6.30E-74	203	Cell activation
4.50E-71	188	Leukocyte activation
6.90E-58	128	Myeloid leukocyte activation
1.70E-54	165	Immune effector process
3.50E-51	189	Regulation of immune system process
2.50E-48	120	Leukocyte activation involved in immune response
1.40E-44	125	Leukocyte mediated immunity
1.50E-44	102	Leukocyte degranulation

b

GO Analysis - Cluster C

adj.Pval	nGenes	Pathways
5.60E-18	14	Muscle filament sliding
5.60E-18	14	Actin-myosin filament sliding
1.30E-17	60	Tissue development
2.60E-15	17	Actin-mediated cell contraction
1.90E-13	17	Actin filament-based movement
4.80E-13	13	Myofibril assembly
7.30E-12	31	Actin filament-based process
9.30E-12	40	Epithelium development
1.00E-11	31	Epithelial cell differentiation
1.00E-11	16	Striated muscle cell development

Supplementary Fig. 21. Gene ontology analysis of differentially expressed genes between control, Tig1 and Tig1^{P155A} overexpressing cells. GO enrichment analysis using g:Profiler of the Biological process terms in the 3 groups determined by K-means hierarchical clustering (number of groups was chosen based on tSNE and Elbow method), displaying the top ten enriched terms for cluster B (a) and C (b). The enriched pathways are listed with their corresponding adjusted p-value and number of genes. The p-values are corrected for multiple testing using False Discovery Rate (FDR).

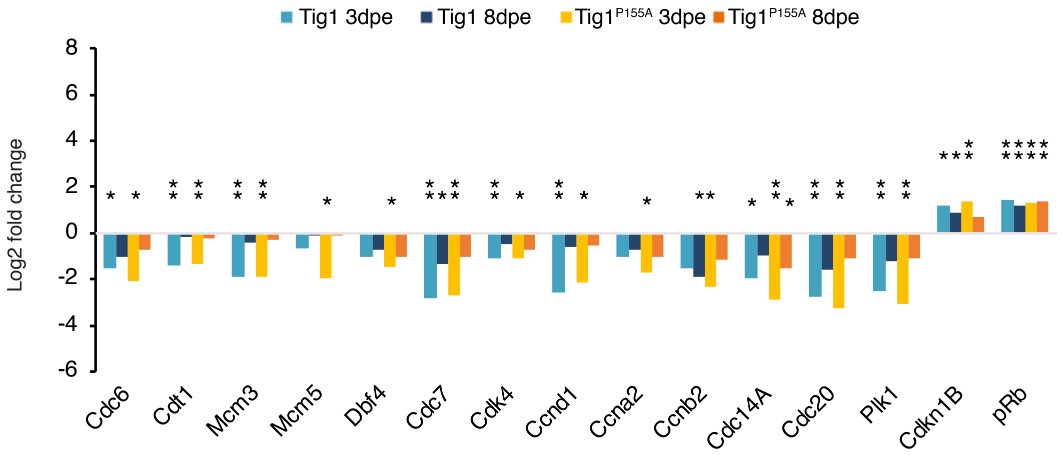

Supplementary Fig. 22. Tig1 overexpression promotes the downregulation of cell cycle progression genes and upregulation of tumour suppressors. Relative gene expression changes in genes associated with cell cycle progression among differentially expressed genes between control, Tig1 or Tig1^{P155A} overexpressing distal cells at 3 or 8 dpe. * p<0.05; ** p<0.001. Source data (including exact p-values) are provided as a Source Data file.

gene	Forward (5' primer)	Reverse (3'primer)
Rlp4	TGAAGAACTTGAGGGTCATGG	CTTGGCGTCTGCAGATTTTTT
Hoxa13	ACTGGTTCATGAAGACAATTGCCT	CTGTTTTCACCGTCTTCCCCATTA
Hoxa11	GCCAAACAGGCTTGGTTTTA	CTTCCGGCGATTGTTTAGTC
Hoxa9	GAGACAAGCCTGCCATTGAC	GGTTCTGGAACCAGATCTTGAC
Meis1	GCCGTCGCCAACAATGATTT	GTGATGCGTACGGTTGATGC
Enpp2	CGATACCAGTCCCAACGCAT	GAGTTGCTCAATGTCCCGGA
Epha7	CGTTACAATCGGCATGAGGG	GCTAGGCCTTACTTCCCGTG
Lhx2	CCTCTTTCAAACACCACCAGTTG	TGTGTTTTCTTGGCGTAGGAGAT
Prod1	GATCTCAAAGGTCGGTTCAGA	CACAAGCCTGCTACTTCTAGA
Tig1CDS	TCCAATGAGCCCCTGAGAGA	GACACTCTTCACTTGGGCCA
Tig1-3'UTR	CTCCCTCAAACAATCCGGCT	GTTGTCGAGCGCTTCCATTC
ef1-α	AACATCGTGGTCATCGGCCAT	GGAGGTGCCAGTGATCATGTT
Tig1 N.vir	AAAGTTGGAACGTGCTCTGG	CATTTGCATTGATTGGCTTG
ef1-α N.vir	TAGAGTGCAGGTGACGATCC	AGTCACCAAGTCTGCCATCA

Supplementary Table 1. qRT-PCR primer sequences.
