# Supplementary material for: Tig1 regulates proximo-distal identity during salamander limb regeneration
Source: Nat Commun. 2022 Mar 3;13:1141. doi: 10.1038/s41467-022-28755-1 (PMC8894484; doi:10.1038/s41467-022-28755-1)
Supplement: Supplementary file 3 — Description of Additional Supplementary Files [file 41467_2022_28755_MOESM3_ESM.pdf]

Description of Additional Supplementary Files

File Name: Supplementary Data 1
Description: Single cell transcriptomic analysis of gene contribution to different principal components (Gerber et al dataset).

File Name: Supplementary Data 2
Description: Hoxa13 and Tig1 coexpression at different TPM cutoffs (Gerber et al dataset). Fisher's exact test rules out co-expression (p value > 0.5 for every cutoff); blue and gray circled depict co-expression and lack of coexpression, respectively

File Name: Supplementary Data 3
Description: List of genes correlated with proximal or distal compartments after PD score (Lin et al, 2021 dataset[19]).

File Name: Supplementary Data 4
Description: Predicted regulatory elements (RA-related, Hox and Meis1/2 binding sites) within 40kb upstream of the start site of Tig1 coding sequence.

File Name: Supplementary Data 5
Description: Elisa and SDS-PAGE analysis of purified antibodies against Tig1 peptide 154

File Name: Supplementary Data 6
Description: Elisa and SDS-PAGE analysis of purified antibodies against Tig1 peptide 155

File Name: Supplementary Data 7
Description: List of up and downregulated genes in Hoxa13/mCherry^{+} cells following Tig1 or Tig1^{P155A} overexpression at 3 and 8dpa

File Name: Supplementary Data 8
Description: List of genes corresponding to the GO pathway enrichment analysis in Fig 6d and Supp. Fig 18 and adjusted p-values.

File Name: Supplementary Data 9
Description: List of Tig1 upregulated genes used for the upregulation score (Fig. 8).

File Name: Supplementary Data 10
Description: List of Tig1 downregulated genes used for the downregulation score (Fig. 8).

File Name: Supplementary Data 11
Description: FACS sample gating strategy (Fig. 6)
