# Supplementary material for: Tig1 regulates proximo-distal identity during salamander limb regeneration
Source: Nat Commun. 2022 Mar 3;13:1141. doi: 10.1038/s41467-022-28755-1 (PMC8894484; doi:10.1038/s41467-022-28755-1)
Supplement: Supplementary file 8 — Supplementary data 5 [file 41467_2022_28755_MOESM8_ESM.pdf]

Eurogentec
Experience true partnership

ANTIBODY SERVICE

ANTIBODY PURIFICATION ANALYSIS BY INDIRECT ELISA

Customer : **Dr. YUN MAXIMINA**

Peptide :	EP104154	*S:*	Serum
Rabbit	1499	*FT:*	Flow-through
		PA:	Purified antibodies

2- Results

		Against Peptide									Against Carrier			
		serum (S)			col. flow-through1 (FT)			purif. Antibodies (PA)			S	FT	PA	
Dilution (x-fold)		1	2	3	4	5	6	7	8	9	10	11	12	
100	A	3,118	3,136	3,121	0,382	0,357	0,400	3,240	3,437	3,498	3,815	3,802	1,158	
300	B	1,527	1,499	1,281	0,366	0,354	0,369	2,427	2,545	2,985	3,771	3,782	0,657	
900	C	0,712	0,608	0,546	0,342	0,333	0,249	1,109	1,269	1,559	3,770	3,769	0,490	
2700	D	0,490	0,354	0,320	0,256	0,292	0,249	0,719	0,586	0,779	3,727	3,733	0,392	
8100	E	0,408	0,321	0,279	0,235	0,260	0,239	0,438	0,459	0,503	3,562	3,447	0,306	
24300	F	0,435	0,281	0,317	0,231	0,241	0,232	0,375	0,415	0,397	3,025	2,857	0,308	
72900	G	0,285	0,280	0,367	0,219	0,237	0,206	0,318	0,360	0,363	0,064	0,062	0,055	- contr.
218700	H	0,266	0,223	0,337	0,204	0,234	0,205	0,299	0,353	0,295	3,693	3,686	3,695	+ contr.

Response against Peptide

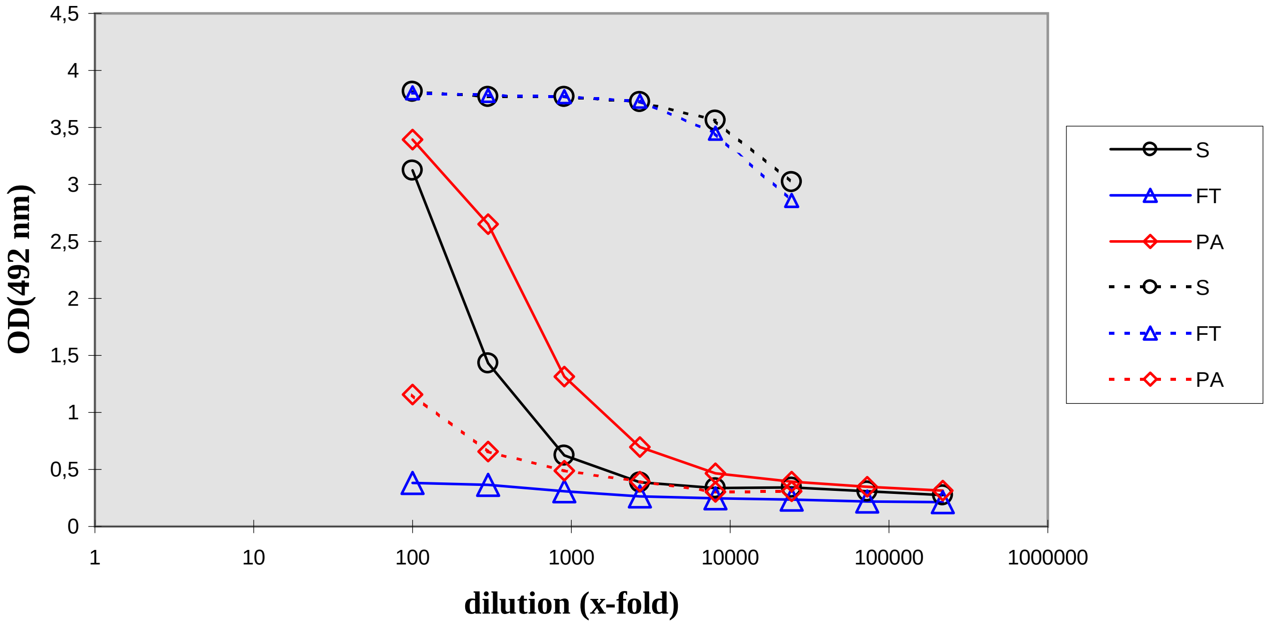

Peptide (straight line) carrier (discontinous line)

Visit **www.eurogentec.com/ELISA-interpretation.html** for ELISA results interpretation

Eurogentec
Experience true partnership

ANTIBODY SERVICE

SDS-PAGE ANALYSIS (Bioanalyser, Agilent)

Customer : **Dr. YUN MAXIMINA**

Peptide : EP104154

Rabbit 1499

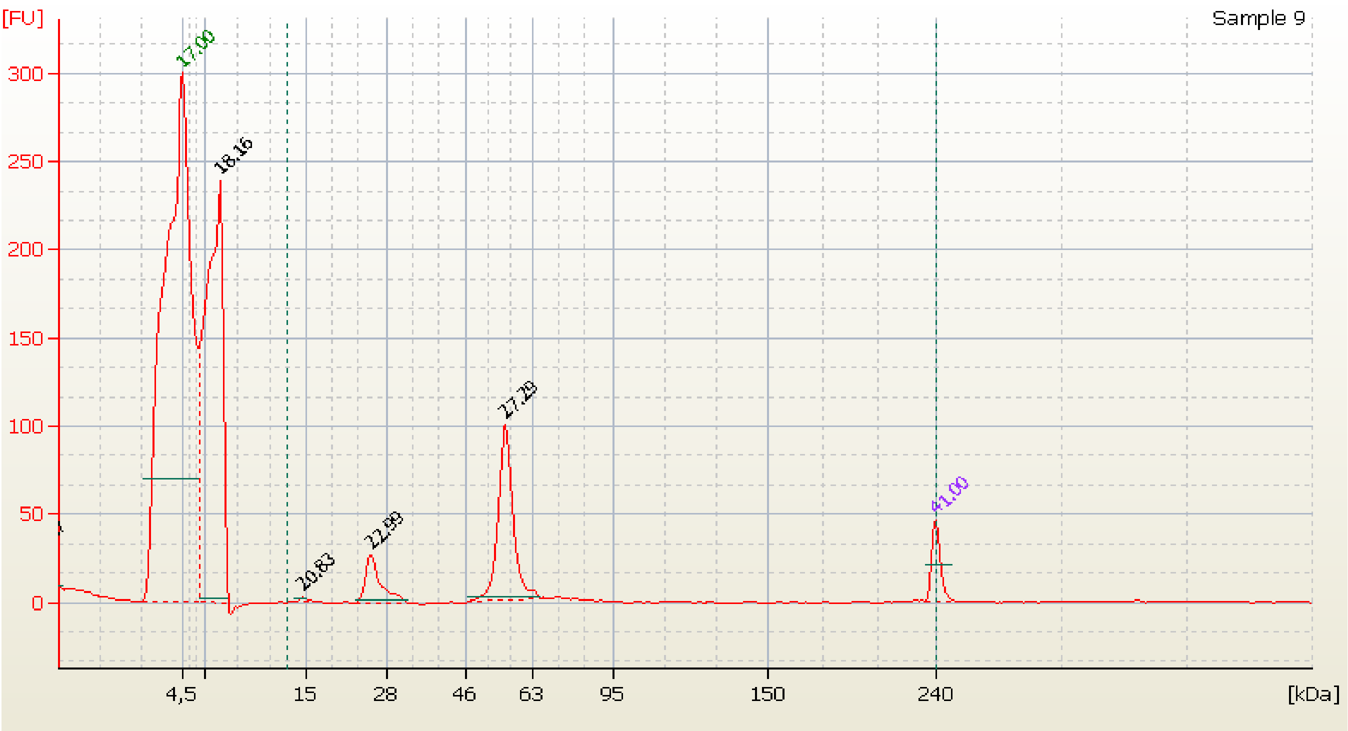

Size [kDa]	% Total	Observations
4,5	0,0	Lower Marker
8,2	0,0	System Peak
14,8	1,3	
25,5	23,7	LC
56,4	75,0	HC
240,0	0,0	Upper Marker

Purity (sum HC+LC) **98,7 %**

LC:	Light Chain
HC:	Heavy Chain
rSA:	rabbit Serum albumin
