# Supplementary material for: Tig1 regulates proximo-distal identity during salamander limb regeneration
Source: Nat Commun. 2022 Mar 3;13:1141. doi: 10.1038/s41467-022-28755-1 (PMC8894484; doi:10.1038/s41467-022-28755-1)
Supplement: Supplementary file 9 — Supplementary data 6 [file 41467_2022_28755_MOESM9_ESM.pdf]

Eurogentec
Experience true partnership

ANTIBODY SERVICE

ANTIBODY PURIFICATION ANALYSIS BY INDIRECT ELISA

Customer : **Dr. YUN MAXIMINA**

Peptide :	EP104155	*S:*	Serum
Rabbit	1499	*FT:*	Flow-through
		PA:	Purified antibodies

2- Results

		Against Peptide									Against Carrier			
		serum (S)			col. flow-through1 (FT)			purif. Antibodies (PA)			S	FT	PA	
Dilution (x-fold)		1	2	3	4	5	6	7	8	9	10	11	12	
100	A	3,460	3,461	3,417	0,712	0,949	0,725	3,664	3,681	3,661	3,805	3,781	0,994	
300	B	2,361	2,236	2,219	0,471	0,491	0,425	3,632	3,654	3,662	3,790	3,758	0,770	
900	C	1,201	0,984	1,036	0,421	0,489	0,424	2,992	3,085	3,128	3,764	3,758	0,558	
2700	D	0,733	0,626	0,588	0,394	0,439	0,422	1,788	1,809	1,910	3,738	3,715	0,472	
8100	E	0,635	0,616	0,396	0,382	0,398	0,390	0,886	0,885	0,944	3,658	3,544	0,448	
24300	F	0,486	0,399	0,393	0,349	0,381	0,382	0,572	0,556	0,538	3,395	3,169	0,359	
72900	G	0,376	0,381	0,389	0,347	0,331	0,330	0,319	0,370	0,326	0,058	0,053	0,062	- contr.
218700	H	0,279	0,259	0,262	0,280	0,283	0,329	0,329	0,287	0,298	3,646	3,621	3,631	+ contr.

Response against Peptide

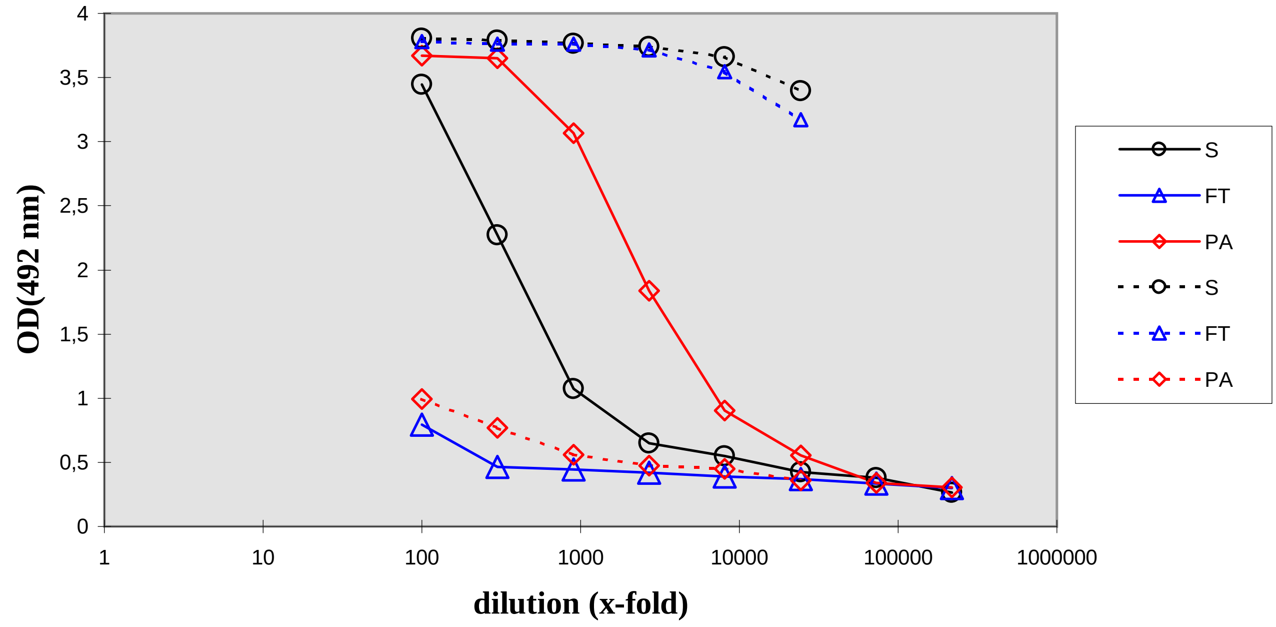

Peptide (straight line) carrier (discontinous line)

Visit **www.eurogentec.com/ELISA-interpretation.html** for ELISA results interpretation

Eurogentec
Experience true partnership

ANTIBODY SERVICE

SDS-PAGE ANALYSIS (Bioanalyser, Agilent)

Customer :	**Dr. YUN MAXIMINA**

Peptide :	EP104155
Rabbit	1499

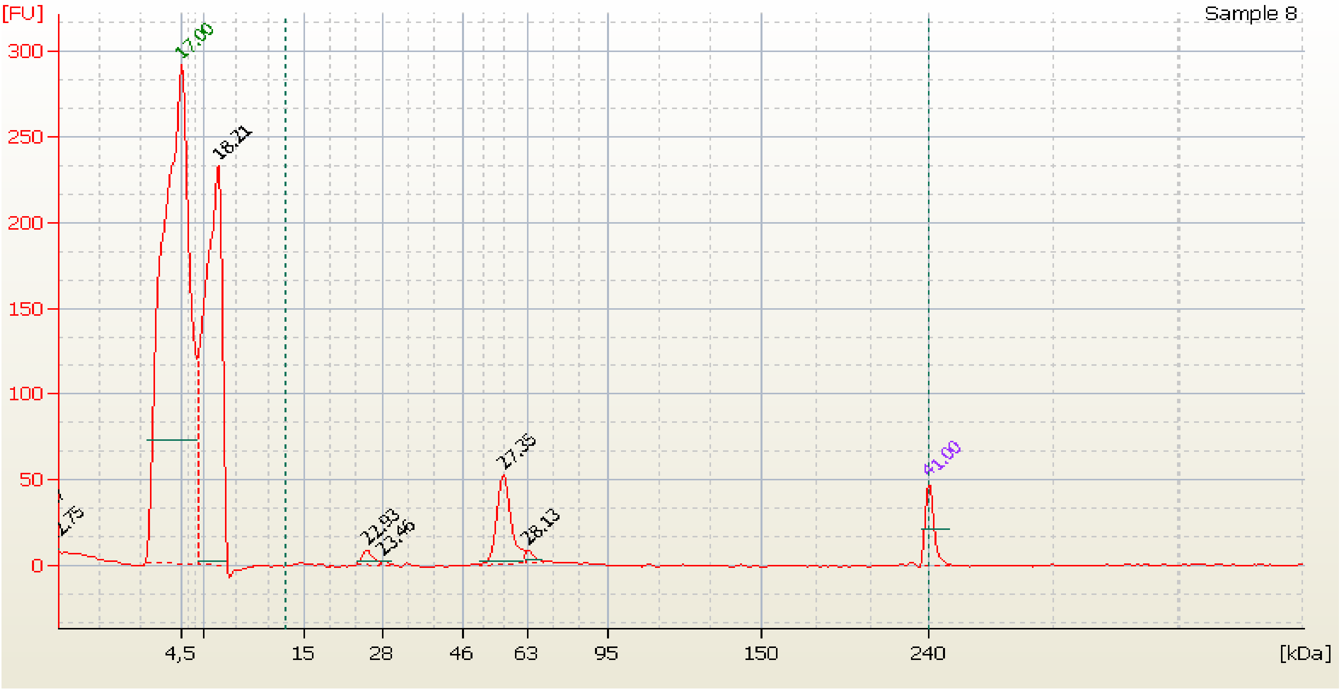

Size [kDa]	% Total	Observations
4,5	0,0	Lower Marker
8,3	0,0	System Peak
25,3	13,0	LC
28,0	2,2	LC
56,8	79,1	HC
63,2	5,7	
240,0	0,0	Upper Marker

Purity (sum HC+LC) **94,3 %**

LC:	Light Chain
HC:	Heavy Chain
rSA:	rabbit Serum albumin
