# Supplementary figures and images for: Tig1 regulates proximo-distal identity during salamander limb regeneration
Source: Nat Commun. 2022 Mar 3;13:1141. doi: 10.1038/s41467-022-28755-1 (PMC8894484; doi:10.1038/s41467-022-28755-1)

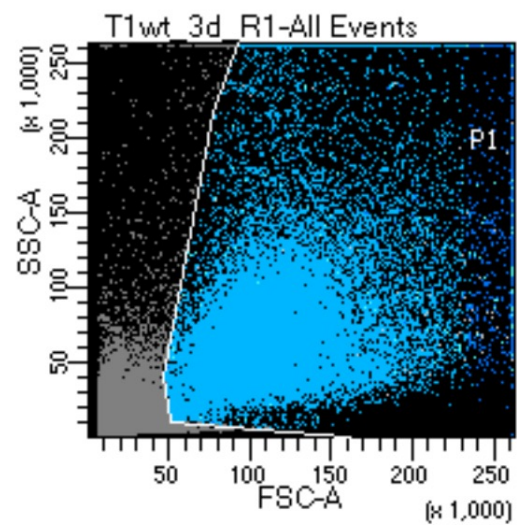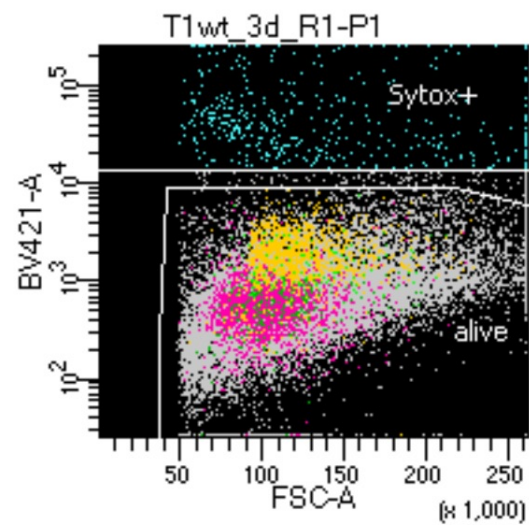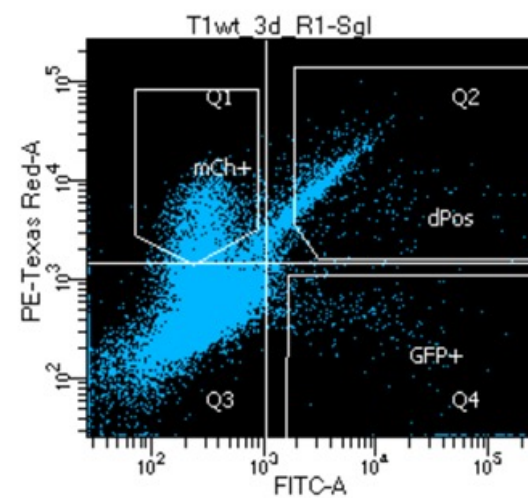

Tube: T1wt\_3d\_R1

| Population | #Events | %Parent | %Total |
|------------|---------|---------|--------|
| All Events | 88,933  | ####    | 100.0  |
| P1         | 33,220  | 37.4    | 37.4   |
| alive      | 32,583  | 98.1    | 36.6   |
| Sgl        | 31,475  | 96.6    | 35.4   |
| Q1         | 3,213   | 10.2    | 3.6    |
| Q2         | 1,779   | 5.7     | 2.0    |
| Q3         | 26,003  | 82.6    | 29.2   |
| Q4         | 480     | 1.5     | 0.5    |
| dPos       | 1,078   | 3.4     | 1.2    |
| mCh+       | 2,386   | 7.6     | 2.7    |
| GFP+       | 278     | 0.9     | 0.3    |
| autofl?    | 895     | 2.8     | 1.0    |
| Sytox+     | 442     | 1.3     | 0.5    |

Supplement: Supplementary file 14 — Supplementary data 11 [file 41467_2022_28755_MOESM14_ESM.pdf]
