# Supplementary material for: Tig1 regulates proximo-distal identity during salamander limb regeneration
Source: Nat Commun. 2022 Mar 3;13:1141. doi: 10.1038/s41467-022-28755-1 (PMC8894484; doi:10.1038/s41467-022-28755-1)
Supplement: Supplementary file 15 — Reporting Summary [file 41467_2022_28755_MOESM15_ESM.pdf]

nature portfolio

Corresponding author(s): Dr Maximina Yun

Last updated by author(s): 13/12/2021

Reporting Summary

Nature Portfolio wishes to improve the reproducibility of the work that we publish. This form provides structure for consistency and transparency in reporting. For further information on Nature Portfolio policies, see our Editorial Policies and the Editorial Policy Checklist.

Statistics

For all statistical analyses, confirm that the following items are present in the figure legend, table legend, main text, or Methods section.

n/a	Confirmed	
☐	☒	The exact sample size (*n*) for each experimental group/condition, given as a discrete number and unit of measurement
☐	☒	A statement on whether measurements were taken from distinct samples or whether the same sample was measured repeatedly
☐	☒	The statistical test(s) used AND whether they are one- or two-sided<br>*Only common tests should be described solely by name; describe more complex techniques in the Methods section.*
☐	☒	A description of all covariates tested
☐	☒	A description of any assumptions or corrections, such as tests of normality and adjustment for multiple comparisons
☐	☒	A full description of the statistical parameters including central tendency (e.g. means) or other basic estimates (e.g. regression coefficient) AND variation (e.g. standard deviation) or associated estimates of uncertainty (e.g. confidence intervals)
☐	☒	For null hypothesis testing, the test statistic (e.g. *F*, *t*, *r*) with confidence intervals, effect sizes, degrees of freedom and *P* value noted<br>*Give P values as exact values whenever suitable.*
☐	☒	For Bayesian analysis, information on the choice of priors and Markov chain Monte Carlo settings
☐	☒	For hierarchical and complex designs, identification of the appropriate level for tests and full reporting of outcomes
☒	☐	Estimates of effect sizes (e.g. Cohen's *d*, Pearson's *r*), indicating how they were calculated

Our web collection on statistics for biologists contains articles on many of the points above.

Software and code

Policy information about availability of computer code

Data collection	No software was used for data collection
Data analysis	In this manuscript we used 'Meandros', an custom image analysis software for which a description is available in the methods section. A separate paper will be published elsewhere and the software will be uploaded to a public repository. The code is available to the Reviewers/ Editor upon request. All other software used is described within the submitted manuscript.<br>Commercially/publicly available software/code used: Seurat package (v4); 'EnhancedVolcano' R package; R package 'rstatix' and 'ggpubr', https://molotool.autosome.ru/; STAR (v2.7.6a; PMID:27115637) ; featureCounts (v2.0.1; PMID:30783653); DESeq2 package; iDEP.93 web application; g:profiler; Prism 4.0 software; Opticon 3 (Bio-rad) software, Zen 2.3 (blue Edition, Zeiss) and Improvision Openlab (PerkinElmer) software.

For manuscripts utilizing custom algorithms or software that are central to the research but not yet described in published literature, software must be made available to editors and reviewers. We strongly encourage code deposition in a community repository (e.g. GitHub). See the Nature Portfolio guidelines for submitting code & software for further information.

March 2021

Data

Policy information about availability of data

All manuscripts must include a data availability statement. This statement should provide the following information, where applicable:

- Accession codes, unique identifiers, or web links for publicly available datasets
- A description of any restrictions on data availability
- For clinical datasets or third party data, please ensure that the statement adheres to our policy

A data availability statement is provided in the manuscript under a separate 'data availability' section. All data generated or analysed during this study are either included in this published article (and its supplementary information files) or publicly available (Gerber et al, Science, 2018 and Lin et al, Developmental Cell, 2021). The raw RNAseq data generated within this study has been deposited in the Gene Ontology Omnibus (GEO)/NCBI database under accession code GSE184948 (https://www.ncbi.nlm.nih.gov/geo/query/acc.cgi?acc=GSE184948). Additional scRNA seq and bulk RNAseq analysis and well as Tig1 antibody generation and validation information is presented as Supplementary Data. Source data are provided with this paper.

Field-specific reporting

Please select the one below that is the best fit for your research. If you are not sure, read the appropriate sections before making your selection.

[x] Life sciences [ ] Behavioural & social sciences [ ] Ecological, evolutionary & environmental sciences

For a reference copy of the document with all sections, see nature.com/documents/nr-reporting-summary-flat.pdf

Life sciences study design

All studies must disclose on these points even when the disclosure is negative.

Sample size	The experimental designs were based on known biological variance of the culture and animal experiments as well as statistical feasibility. Power analysis was performed for sample size estimation using G*Power software. For our in vivo work, prior experience on the various assays was taken into account to achieve appropriate cohort size whilst minimising animal use. For the engulfment assay, sample size was determined considering Da Silva et al, Dev Cell 2002. For displacement assay, sample size determination took into consideration experiments from Echeverri & Tanaka, Dev Biol 2005. Assays involving Baculovirus delivery took into account Oliveira et al, Dev Biol 2018. For the RNAseq experiment, sample size was calculated based on the electroporation efficiency and the number of cells required for the sequencing protocol.
Data exclusions	No data were excluded from our analyses, with one exception: any images/part of images that did not reach the high-resolution standards (e.g. due to blurriness or suboptimal signal detection) required for Meandros analysis were excluded.
Replication	To ensure the reproducibility of experimental findings we performed an appropriate number of technical and biological replicates. Independent experiments were used to verify our findings, the number is indicated in the corresponding figure legend. Each experiment was independently repeated at least three times. All replication attempts ere successful.
Randomization	Randomised sample allocation was used for all the described experiments.
Blinding	Blinding was used for scoring of the engulfment, EdU, segment length estimation upon baculovirus transduction, scratch and displacement assays.

Reporting for specific materials, systems and methods

We require information from authors about some types of materials, experimental systems and methods used in many studies. Here, indicate whether each material, system or method listed is relevant to your study. If you are not sure if a list item applies to your research, read the appropriate section before selecting a response.

Materials & experimental systems

n/a	Involved in the study	
[ ]	[x]	Antibodies
[ ]	[x]	Eukaryotic cell lines
[x]	[ ]	Palaeontology and archaeology
[ ]	[x]	Animals and other organisms
[x]	[ ]	Human research participants
[x]	[ ]	Clinical data
[x]	[ ]	Dual use research of concern

Methods

n/a	Involved in the study	
[x]	[ ]	ChIP-seq
[x]	[ ]	Flow cytometry
[x]	[ ]	MRI-based neuroimaging

Antibodies

Antibodies used	Polyclonal anti-TIG1 antibodies (custom for axolotl Tig1, specified in the manuscript, validated through ELISA -suppl data-, antibody staining and neutralisation of function - this manuscript-)<br>anti-PROD1 (custom for axolotl Prod1 -Da Silva et al, 2002-)<br>anti-PRRX1 (custom for axolotl PRRX1 -Gerber et al, 2008-)<br>anti-TMEFF1 (custom antibodies against EGF-like domain of TMEFF1, see Da Silva et al, 2002 and Da Silva et al Mech Dev 2001)<br>Anti-Myc (mouse monoclonal, clone 9E10, Sigma-Aldrich, cat no. M4439, LOT #110M4770)<br>anti-RFP (rabbit polyclonal, Rockland, Cat no.600-401-379, LOT #45808)
Validation	Validation of the anti-axolotl Tig1 antibodies was performed by ELISA, Western blotting (eg appearance of band of right molecular weight and only upon expression of axolotl Tig1 upon trasnfection of human HEK293T cells), neutralization studies (scratch assay, engulfment assay) in this manuscript. Validation of all other antibodies is available elsewhere (literature or manufacturer). In particular:<br>anti-PROD1 (custom for axolotl Prod1, validated elsewhere -Da Silva et al, 2002-)<br>anti-PRRX1 (custom for axolotl PRRX1, validated elsewhere -Gerber et al, 2008-)<br>anti-TMEFF1 (custom antibodies against EGF-like domain of TMEFF1, validated by Da Silva et al Mech Dev 2001, including exogenous expression of salamander TMEFF1 in COS7 cells and specific reactivity of antibody after immunostaining of transfected cells)<br>Commercial antibodies used in this manuscript were further validated based on their performance against experimental controls as well as molecular weight ladders.

Eukaryotic cell lines

Policy information about cell lines

Cell line source(s)	A1 (Notophthalmus viridescens, gift from Jeremy Brockes); AL1 (Ambystoma mexicanum, gift from Stephane Roy); HEK293T (Homo sapiens, gift from Jeremy Brockes); SF9 ESF (Sodoptera frugiperda, expresSF+, ProteinSciences Corporation; sourced from MPI CBG Protein Expression Facility).
Authentication	All cell lines used are published. A1 and AL1 are custom, non-commercial cell lines developed by others in the field and shared across the community. We did not formally authenticate them, but both lines always exhibited features previously attributed to each of them (e.g. myogenic capacity of newt A1 line, capacity of A1 or AL1 cells to integrate with regenerating salamander tissues, etc). HEK293T were a gift from Jeremy Brockes, not authenticated by our lab. SF9 line was authenticated in Oliveira et al, Dev Biol 2018.
Mycoplasma contamination	All cell lines are negative for mycoplasma contamination.
Commonly misidentified lines (See ICLAC register)	No commonly misidentified cell lines were used in this study.

Animals and other organisms

Policy information about studies involving animals; ARRIVE guidelines recommended for reporting animal research

Laboratory animals	This study involves use of axolotls (Ambystoma mexicanum) and red-spotted newts (Notophthalmus viridescens). Axolotls of the leucistic (d/d) strain were used in all experiments, except for a) the engulfment assay, where Caggs:EGFP47 transgenics were used, and b) the distal cell reprogramming assay, where Hoxa13:Hoxa13-T2a-mCherry)Etnka axolotls were used. Animals between 3 and 13cm length snout-to-cloaca we used, as specified in legends and methods where relevant. A 50:50 ratio of males/females was used in all experiments.
Wild animals	No wild animals were used in the study.
Field-collected samples	No field collected samples were used in the study.
Ethics oversight	Procedures for care and manipulation of all animals used in this study were performed in compliance with the Animals -Scientific Procedures- Act 1986 (United Kingdom Home Office), and the laws and regulations of the State of Saxony, Germany.

Note that full information on the approval of the study protocol must also be provided in the manuscript.
